# Evolutionarily conservative and non-conservative regulatory networks during primate interneuron development revealed by single-cell RNA and ATAC sequencing

**DOI:** 10.1038/s41422-022-00635-9

**Published:** 2022-03-10

**Authors:** Ziqi Zhao, Dan Zhang, Fuqiang Yang, Mingrui Xu, Shaoli Zhao, Taotao Pan, Chuanyu Liu, Yongjie Liu, Qingfeng Wu, Qiang Tu, Ping Zhou, Rong Li, Jia Kang, Lan Zhu, Fei Gao, Yaqing Wang, Zhiheng Xu

**Affiliations:** 1 State Key Laboratory of Molecular Developmental Biology, CAS Center for Excellence in Brain Science and Intelligence Technology, School of Future Technology, Institute of Genetics and Developmental Biology, Chinese Academy of Sciences, Beijing, China; 2 University of Chinese Academy of Sciences, Beijing, China; 3 BGI-Beijing, Beijing, China; 4 BGI-Shenzhen, Shenzhen, China; 5 State Key Laboratory of Molecular Developmental Biology, Institute of Genetics and Developmental Biology, Innovation Academy for Seed Design, Chinese Academy of Sciences, Beijing, China; 6 Key Laboratory of Genetic Network Biology, Institute of Genetics and Developmental Biology, Chinese Academy of Sciences, Beijing, China; 7 Center for Reproductive Medicine, Department of Obstetrics and Gynecology, Peking University Third Hospital, Beijing, China; 8 Department of Obstetrics and Gynecology, Peking Union Medical College Hospital, Beijing, China; 9 State Key Laboratory of Reproductive Biology, Institute of Zoology, Chinese Academy of Sciences, Beijing, China

**Keywords:** Transdifferentiation, Genome-wide association studies

## Abstract

The differences in size and function between primate and rodent brains, and the association of disturbed excitatory/inhibitory balance with many neurodevelopmental disorders highlight the importance to study primate ganglionic eminences (GEs) development. Here we used single-cell RNA and ATAC sequencing to characterize the emergence of cell diversity in monkey and human GEs where most striatal and cortical interneurons are generated. We identified regional and temporal diversity among progenitor cells which give rise to a variety of interneurons. These cells are specified within the primate GEs by well conserved gene regulatory networks, similar to those identified in mice. However, we detected, in human, several novel regulatory pathways or factors involved in the specification and migration of interneurons. Importantly, comparison of progenitors between our human and published mouse GE datasets led to the discovery and confirmation of outer radial glial cells in GEs in human cortex. Our findings reveal both evolutionarily conservative and nonconservative regulatory networks in primate GEs, which may contribute to their larger brain sizes and more complex neural networks compared with mouse.

## Introduction

The complex and orderly neural network of human brain is mainly comprised of excitatory glutamate neurons and ɣ-aminobutyric acid-containing (GABAergic) inhibitory interneurons.^1–3^ Interneurons are highly diverse neurons with the characteristics of different morphologies, connectivity and biological functions.^4–8^ Disturbance of excitatory/inhibitory balance is associated with many neurodevelopmental and neuropsychiatric disorders, such as autism spectrum disorder and schizophrenia.^9–12^ During brain development, only a few interneurons originate and function at local regions, while almost all the rest of interneurons are derived from the ganglionic eminences (GEs), the transitory structures during the embryonic stage.^13–15^ The GEs, located between the thalamus and caudate nucleus, are the origins of interneurons. GEs can be divided into three subregions, including lateral, medial and caudal ganglionic eminences (LGE, MGE and CGE).^14–17^ Each subregion expresses specific genes and generates temporally and spatially distinct interneurons targeted to the whole brain. The MGE mainly generates the GABAergic cells migrating to the cerebral cortex, striatum and globus pallidus, which are characterized by the expression of *NKX2-1* and *LHX6.*^17–21^ The LGE derived interneurons mainly comprise the precursors of the striatal medium spiny neurons (MSNs) and olfactory bulb interneurons, which can be identified by the expression of *ISL1* and *CHD7* respectively.^22–26^ The CGE mainly produces 5HT3aR^+^ cortical interneurons with the specific expression of *NR2F1* and *NR2F2.*^5,18,27,28^ Specific expression of genes and regulatory networks during GE development contribute to the regional cell diversity and interneuron migration.^29–31^

Single cell RNA-seq (scRNA-seq) has been applied in the researches of developmental neuroscience.^32–34^ Compared with traditional bulk RNA-seq, which generates the mixed expression data from tissues, scRNA-seq can provide transcriptional profiles of single cells.^35^ Technology with such a high resolution can distinguish different cell types and facilitate our investigation of the complexed neural system.^36^ Several single cell studies in mice have revealed the diversity of molecular and transcriptional characteristics of GE interneurons during embryonic development.^17,37–39^ However, only a few studies have been reported for the generation of inhibitory neurons during GE development in primate, and the conservation and heterogeneities between rodents and primates are still limited due to the lack or quality of transcriptional analysis.^40^ In addition, the complicated regulatory networks underlying the sophisticated cell proliferation, spatial distinction, lineage determination and interneuron migration are still not clear.^41,42^

In this study, we used the scRNA-seq and single cell Assay for Targeting Accessible-Chromatin with high-throughput sequencing (scATAC-seq), a technology which enables the capturing of open regions on chromatins,^43,44^ to investigate the transcriptional trajectories profiles of GEs in the fetuses of human and *Macaca fascicularis*. We found that most of the gene regulatory networks were well conserved in the developing GEs between human and macaque, including those involved in cell proliferation, cell fate determination and migration. We identified those genes that are potentially important for cell-fate determination in different human GE regions. Moreover, we discovered novel regulatory pathways that may be involved in the fate determination of MGE and LGE, and cell migration in GEs. Importantly, the existence of outer radial glial cells was confirmed in human developing GEs.

## Results

### Cell diversity in the early developmental stages of primate GEs

**Fig. 1:**
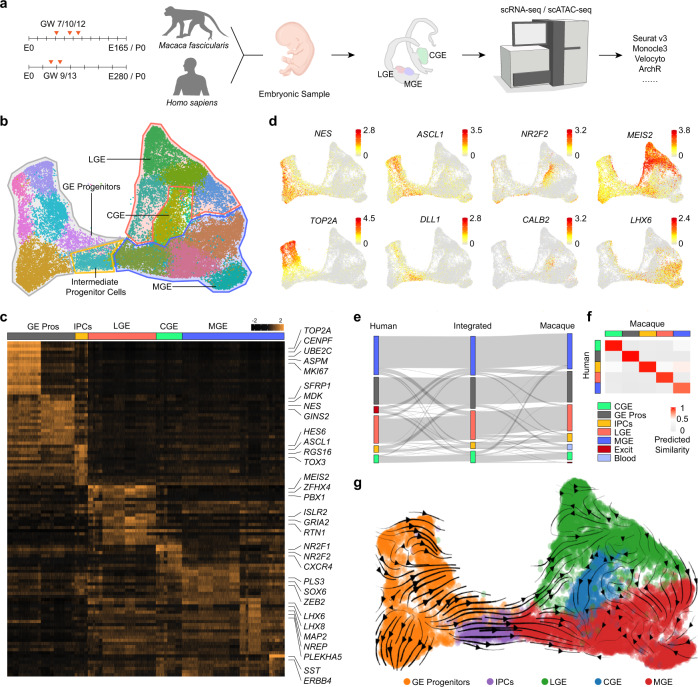
Transcription profiles of embryonic primate GEs. **a** Schematic overview of the workflow. Embryonic samples were collected and prepared for scRNA-seq. Different types of software were applied for downstream analysis. **b** Cell clustering of integrated primate data visualized by UMAP. Clusters were further manually grouped into different colorful panels and annotations were added according to the expression of marker genes shown in **b**, **c**. **c** Heatmaps depicting conserved genes enriched in different cell types from fetal primate GEs. **d** Expression profiles of well-recognized marker genes in progenitors, IPCs, MGE, LGE and CGE, visualized by UMAP. **e** Riverplot illustrating the corresponded relationship between human and macaque data. Integrated data in the middle represented the annotated groups in **a**. A small number of misplaced cells (e.g. excitatory cells in MGE of integrated data) were corrected hereinafter. Legends are listed on the right. **f** Similarity analysis of gene expression in **e**. **g** Gene velocity flow map visualized on UMAP embedding of cell clusters. Most naive sites were located in GE progenitors. Streamlines represented the RNA velocity predicting the future transcriptional dynamic state of cells.

We preformed droplet-based scRNA-seq of GEs from three *Macaca fascicularis* and two human fetuses in order to dissect and compare the developmental profile of primate GEs. Macaque samples were from gastral week (GW) 7, 10 and 12 when GEs are still in the early stage of development. Human samples were collected from GW 9 and 13 which are likely in the comparable developmental windows (Fig. 1a). After sequencing, mapping and quality control, we got 13,782 cells from human and 29,269 cells from macaque (Supplementary information, Table S1). Then the data from two species were integrated by Seurat3.0.^45^ Unsupervised method was used to cluster different cells (Fig. 1b), and we detected 21 clusters of cells (Supplementary information, Fig. S1a). Each clusters were annotated by well-known markers for the classification of those cells (Fig. 1b). We removed two clusters of excitatory neurons from neighboring cerebral cortex expressing *NRN1* and *SLA*^46,47^ (Supplementary information, Fig. S1b, c). We also found the clusters of microglia and hemocytes discerned by their corresponding markers, *CX3CR1* and *SLC4A1*^48,49^ (Supplementary information, Fig. S1d), which were also removed. Among the remaining GE derived cells, we could distinguish cells from GE progenitors and post-mitotic cells by differential gene expression analysis (Supplementary information, Fig. S1e). Neural progenitors including radial glia cells (RGCs) and intermediate progenitors (IPCs) of GEs showed high expression levels of *NES, VIM* or *ASCL1, DLL1*^50^ (Fig. 1c, d and Supplementary information, Fig. S1e, f). Proliferating cells in cell cycle could be marked by *TOP2A*^51^ (Fig. 1c, d). Postmitotic cells could be further defined by regional specific markers (Fig. 1c). MGE comprised cells specifically expressing *NKX2-1* and *LHX6*, while *MEIS2*, *ISLR2* are expressed in LGE and *NR2F1*, *NR2F2* in CGE (Fig. 1d and Supplementary information, Fig. S1e, f). We then calculated the normalized cell ratio of human and macaque cells for each cell type, which was visualized in river plot, suggesting that no significant difference between human and macaque GE cell partitions (Fig. 1e and Supplementary information, Fig. S1g). Similarity analysis based on gene expression between different cell types from human and macaque also proved the accuracy in the integration of the data from the two species (Fig. 1f).

To present the trajectory of cell development in primate GEs, we performed RNA velocity analysis (scVelo),^52,53^ a method to infer trajectory by reconstructing the developmental sequence of transcriptional changes. As predicted, cells originated from GE progenitors proliferated and generated IPCs,^54^ and then differentiated into various lineages in MGE, LGE and CGE (Fig. 1g). In conclusion, our analysis confirmed the basic evolutional conservation between macaque and human, and revealed the cell diversity and developmental trajectory in human and macaque GEs.

### Specific cell identities of primate GE progenitors

**Fig. 2:**
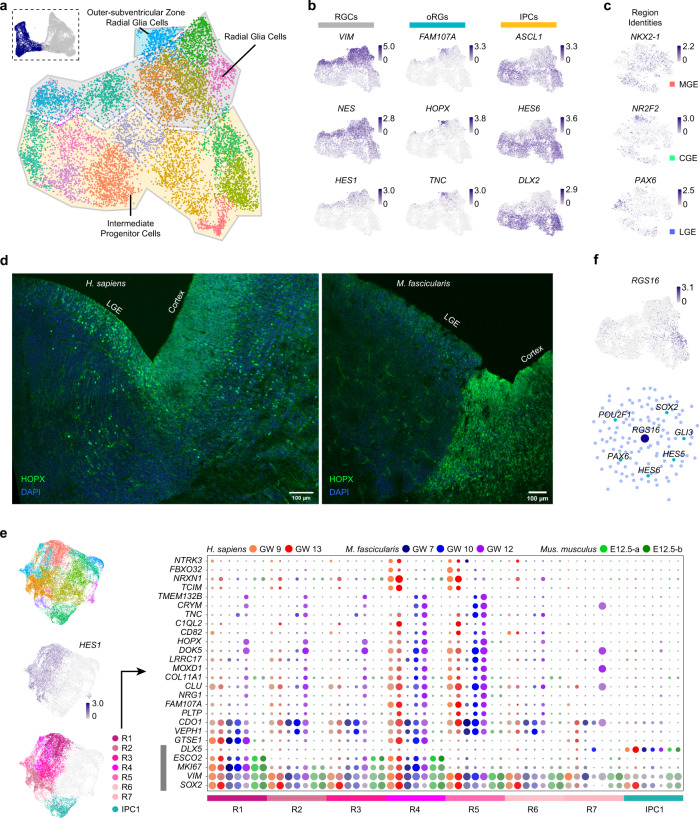
Characterization of primate GE progenitors and the comparison between human and mouse. **a** Progenitor cell groups of primate GEs. Selected clusters of progenitors from integrated data (top left) were further analyzed and divided (right, visualized by UMAP). **b** Expression levels of common markers in different kinds of progenitors. **c** Expression levels of regional identity markers for MGE, CGE and LGE in all RGCs (visualized by t-SNE). **d** Expression of HOPX in human (left) and macaque (right) GE, especially in LGE as well as in cortex. Immunofluorescence staining of HOPX at the intersection of GE and cortex. DAPI labels nuclei. **e** Progenitors of human, macaque and mouse GE visualized by UMAP. *HES1*^+^ RGCs were separated into 7 groups (R1–R7) for differential gene expression analysis across species (left). DEGs across human, macaque and mouse GE progenitors (right). *SOX2*, *VIM*, *MKI67*, *ESCO2* and *DLX5* labeled by gray bar illustrated their progenitor identity. Different colored bars represented the cell clusters on the left panels. Different colors of dot indicated different species. Size of dot represented the percentage of cells in these group expressing certain genes while color saturation represents the expression level. **f** Expression levels and possible upstream regulators of *RSG16*. Expression was visualized by UMAP (top). Upstream regulators were predicted and revealed by gene regulation networks (bottom).

In mammalian developing brain, neurons are generated by the RGCs located in the ventricular zone (VZ) and IPCs in the subventricular zone (SVZ) in the cerebral cortex and GE.^55,56^ We subset the neural progenitor cells from the human and *Macaca fascicularis* GE, including RGCs and IPCs (Fig. 2a). RGCs were distinguished by the high expression levels of *HES1*, *VIM* and *NES*, while IPC was identified by the expression of *ASCL1, HES6* and *DLX2*^50,57^ (Fig. 2b). Among all the RGCs, we observed the regional identities of different GE progenitors. Expression of *NKX2-1*, *PAX6* and *NR2F2* represented the MGE, LGE and CGE progenitors respectively (Fig. 2c).

Studies of the cerebral cortex revealed a specific proliferative region outside the SVZ/VZ, named outer subventricular zone (oSVZ) in the primate neocortex.^56^ Outer radial glia cells (oRGs) located in the oSVZ are different from RGCs in the VZ, and they are considered to be the major contributor of brain volume expansion in primates, especially human.^56^ Notably, we identified the primate specific outer radial glia cells (oRGs) within the RGCs of GEs based on the concentrated and specific expression of *FAM107A, HOPX* and *TNC*^58^ (Fig. 2a, b). We performed immunostaining of HOPX and confirmed the expression of HOPX in both the VZ and SVZ of the GW13 human and GW12 macaca LGE, as well as those areas far away from the VZ, similar to those in the cortex (Fig. 2d). However, we did not observe any positive signals in mouse GE or cortex (Supplementary information, Fig. S2a). This indicates that oRGs exist not only in the cerebral cortex, but also in GEs during the primate brain development.

We then sought to find the differences of GE progenitors between primate and mouse. We integrated our primate data with a published mouse GE dataset (see Method and Material), and subset the progenitor clusters. Focusing on the *HES1*^*+*^ RGCs, we performed differential gene expression analysis between different species (Fig. 2e and Supplementary information, Fig. S2b). A group of genes highly expressed in human and macaque were identified (Fig. 2e and Supplementary information, Fig. S2c). Among those differentially expressed genes (DEGs), some of them were previously reported as specifically expressed genes in human RGCs, such as *MOXD1* and *TCIM*^58–60^ (Fig. 2e and Supplementary information, Fig. S2c), which verified our discovery. Other proliferation related genes such as C*LU*^61^ and *VEPH1,*^62^ might contribute to species differences as well (Fig. 2e and Supplementary information, Fig. S2c).

The transitions from RGCs to IPCs and mitotic to postmitotic cells play important roles in cell development and differentiation during brain development. Analysis of IPCs revealed that *RGS16*, a regulator of G protein-coupled receptor signaling cascades,^50,63,64^ was specifically expressed in those cells that just got into postmitotic period in both primate and mouse (Fig. 2f and Supplementary information, Fig. S2d, e). Gene regulatory network performed by scATAC-seq showed that *RGS16* was regulated by several proliferation related genes, such as *PAX6*, *GLI3* and *HES5*^65–67^ (Fig. 2f). Moreover, regional specific markers, such as *NKX2-1* in MGE, *NR2F2* in CGE and *ISL1* in LGE started to express correlated to the reduced expression of *RGS16* (Supplementary information, Fig. S2d). These patterns suggest that RGS16 may act as a transition factor between IPCs and cell fate-determined precursors.

The above results indicate that oRGs are present in primate in both developing GE and cerebral cortex, which may contribute to the species difference between primates and rodents.

### Cell fate determination in the developing MGE and CGE

**Fig. 3:**
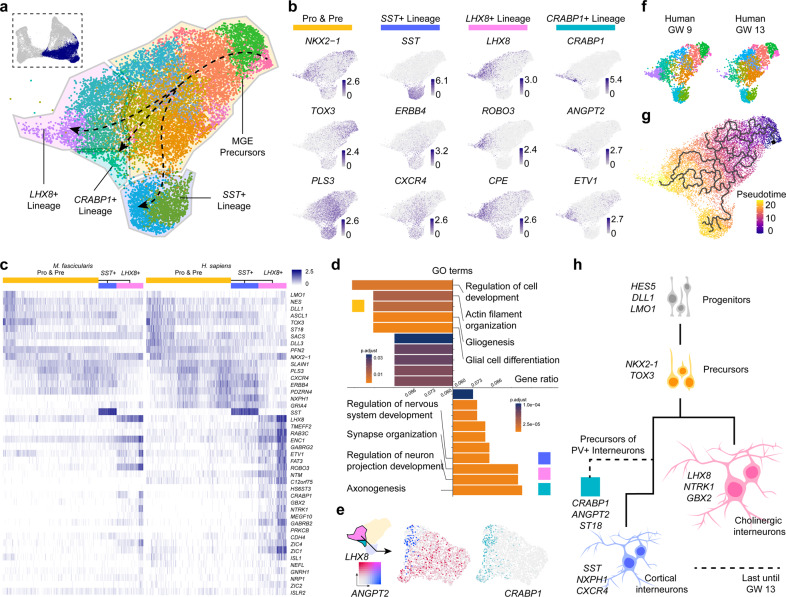
Analysis of primate MGE development. **a** Clusters and cell types in MGE. Cell clusters from MGE were selected (top left) and further characterized (middle, visualized by UMAP). Trajectory was reconstructed and depicted in dash lines based on gene expression pattern and pseudotime analysis. **b** Visualization of expression profiles of different cell lineages in MGE using UMAP. Pro, progenitors; Pre, precursors. **c** Heatmaps describing DEGs in different lineages. *CRABP1*^+^ lineage was not shown in it due to its few counts of the cells. **d** GO enrichment analysis in different cell lineages. Marker genes of MGE progenitors and precursors were selected for GO enrichment analysis (top), as well as marker genes of other lineages (bottom). **e** Exclusive expression pattern of *ANGPT2* and *LHX8*, with relatively enriched expression of *CRABP1* in *ANGPT2* lineage. **f** Human samples of different developmental stages of MGE, with cell numbers of *LHX8*^+^ lineage showing the obvious decrease from GW 9 to GW 13. **g** Pseudotime trajectory of primate MGE. **h** Schematic conclusion for possible primate MGE lineages developmental process. Corresponding marker genes were labeled nearby.

To depict the developmental lineages in MGE, *LHX6-*expressing groups were selected for further differential gene expression analysis and trajectory reconstruction (Fig. 3a and Supplementary information, Fig. S3a). We detected four distinct cell types with one of them presumed to be precursors based on their gene expression profiles. The three other branches specifically comprised *SST-*, *LHX8-* and *CRABP1-*expressing cells respectively (Fig. 3b, c).

MGE precursors were specified by the expression of transcription factors *NKX2-1*, *TOX3*, and *PLS3* (Fig. 3b), which were gradually expressed during cell maturation. All of the presumed more mature cells in MGE shared the expression of *NRXN1* and *CHL1* (Supplementary information, Fig. S3b), whose functions are related to cell adhesion and axon projection according to biological process gene ontology (Fig. 3d).

Throughout the lineage of *SST*^+^ interneurons, *ZEB2* and *PLS3* were expressed along with genes including *ERBB4* and *CXCR4* (Fig. 3b and Supplementary information, Fig. S3b). Previous studies have reported that ERBB4 and CXCR4 regulate the tangential migration of interneurons derived from GE towards cerebral cortex in mice.^68,69^ These patterns suggest that the *SST*^+^ interneurons originated from MGE would migrate to the cerebral cortex.

*LHX8*^+^ cells are MGE-derived cholinergic projection neurons destined to the basal telencephalon. Most of *LHX8*^+^ cells were detected in the MGE of human GW9 and macaque GW7, but barely in the three older samples (Fig. 3f and Supplementary information, Fig. S3c). Similarly, much more *LHX8*^*+*^ cells were detected at E11 and 14 than that at E17 in mouse MGE (Supplementary information, Fig. S3c). This suggests that *LHX8*^+^ cholinergic projection neurons may be generated early in primate brain development, which is consistent with the findings in mouse.

We also found a small group of cells that specifically express *CRABP1* and *ANGPT2* alongside the *LHX8*^+^ cells (Fig. 3b–e). We analyzed those two groups of cells independently in order to find out more details about them. Feature plots revealed that *ANGPT2* expression was completely separated from that of *LHX8*, while *CRABP1* was expressed in a small percentage of *LHX8*^+^ cells (Fig. 3e). Further inspection of DEGs led to the finding of *ST18* and *ETV1* expression in the *ANGPT2*^*+*^ group (Supplementary information, Fig. S3d). *ST18* and *ETV1* were described as the markers of cortical parvalbumin (PV) interneurons precursor.^17^ We assumed that those cells would migrate towards cerebral cortex during brain development and represent the precursors of cortical PV interneurons.

The above results revealed that the cells differentiate early in MGE and the cell lineages diversify in a time-dependent manner, which was also presented in pseudotime analysis constructed by Monocle3^70^ (Fig. 3g, h).

CGE mainly generated *5HT3aR*^+^ cortical interneurons. We extracted the CGE cells to inspect their cellular diversity (Supplementary information, Fig. S3e). Almost all of the CGE cells highly expressed *NR2F1* and *NR2F2* (Supplementary information, Fig. S3f). At this development stage, we detected the expression of *CALB2* and *CCK* (Supplementary information, Fig. S3f), which could represent a group of bipolar and multipolar interneurons in the adult cerebral cortex.^5,18,71^ However, other classic CGE-derived interneurons markers, such as *RELN* and *VIP* were barely expressed in our data (Supplementary information, Fig. S3f). These results suggested that the *CCK*^*+*^ and *CALB2*^*+*^ interneurons might be specified earlier than the *RELN*^*+*^ and *VIP*^*+*^ interneurons in the primate CGE.

### Cell fate decision in the developing LGE

**Fig. 4:**
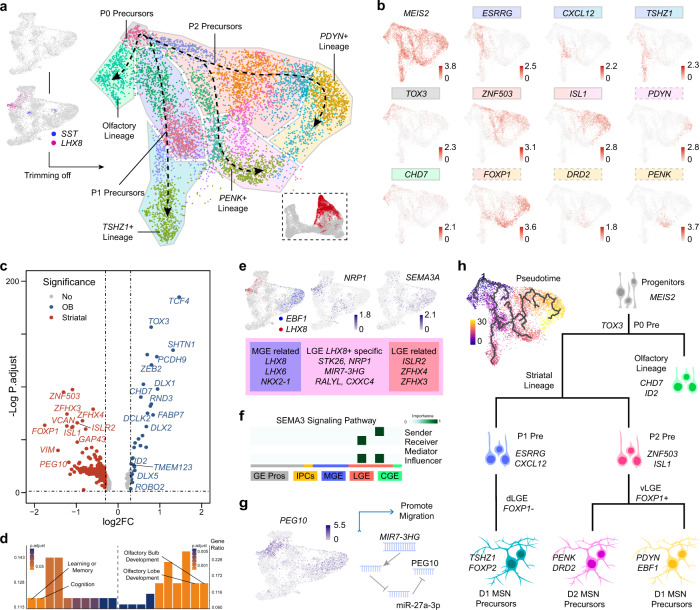
Characterization of LGE development and possible migrating cells. **a** UMAP clusters of cells in LGE. Clusters of LGE from integrated data were selected and *LHX8-* or *SST-*expressing cells were trimmed (left). Remaining cells were marked in UMAP plot (bottom right) and re-clustered (middle). Trajectory was reconstructed and depicted in dash lines based on gene expression pattern and pseudotime analysis. **b** Landscapes of marker genes in various LGE-derived lineages. Background colors of genes were consistent with colors of cell lineage groups in **a**. Dash line of gene name frames represents destiny of corresponding cells to vLGE. **c**. Volcano plots of DEGs that are expressed at high levels in olfactory lineage (right, Olfactory lineage in **a**) or striatal lineages (left, three other striatal lineages in **a**). False discovery rates were adjusted by BH method to generate “P. adjust”. **d** GO enrichment of significant DEGs revealed in **c** in different lineages. False discovery rates were analyzed as in **c**. **e** Gene profiles of LGE *LHX8*^+^ linages. Similar exclusive expression pattern could be found between *LHX8*-*EBF1* gene pair and *NRP1*-*SEMA3A* gene pair (top). Mixed expression feature of LGE *LHX8*^+^ lineage, cell type specific genes were also detected (bottom). **f** SEMA3A signaling pathway prediction provided by CellChat, based on human cells from integrated data. Colored bars represented the cell types defined in Fig. **1a**. **g** *PEG10* as a potential migration regulator. Expression level of *PEG10* (left) and schematic model of one activation pathway of *PEG10* by *MIR7-3HG* (right). **h** Pseudotime trajectory and schematic overview of hypothetic differentiation program. Markers of matched cell types were labeled nearby.

LGE was known as the origin of striatal MSNs and olfactory bulb (OB) interneurons.^22,24^ To reveal its developmental trajectories, we selected the LGE lineage from the primate combined data. After unsupervised clustering, seven different cell types were detected in the primate developing LGE (Fig. 4a and Supplementary information, Fig. S4a). We found that striatal MSN precursors could be divided by the expression of *FOXP1* into dorsal LGE (dLGE) and ventral LGE (vLGE).^72,73^ In dLGE, the majority of cells were the *TSHZ1*^+^ striatonigral (D1) MSNs. In vLGE, there were two types of interneurons, with striatonigral (D1) MSNs expressing *PDYN* and striatopallidal (D2) MSNs expressing *PENK*^74–76^(Fig. 4b).

We then performed differential gene expression analysis between the OB lineage and three other striatal lineages. Volcano plot revealed that the OB lineage cells expressed at high levels of the stem cell related genes, such as *TOX3, CHD7* and *ID2*^77–79^ (Fig. 4c). Gene ontology analysis of those DEGs was in support of the evidence that OB interneuron precursors exist in the LGE (Fig. 4d). The cell distribution at different time showed that most of the OB lineage cells were detected from GE at later stages both in human and macaque (Supplementary information, Fig. S4b). These results suggest that OB interneuron precursors were generated later than striatal MSN precursors in LGE and would then migrate to OB for further proliferating.^23^

Interestingly, we also found a group of cells shared some MGE markers different from the striatal MSNs, such as *LHX8*, *NKX2-1* and *LHX6*, and LGE markers including *ZFHX3* and *ISLR2*^80^ (Fig. 4e and Supplementary information, Fig. S4c). The absence of *MEIS2* expression in those cells further indicated that they were different from LGE derived cells (Supplementary information, Fig. S4c). To figure out the identity and origin of those cells, we found their specific markers that were not expressed in other typical LGE or MGE clusters (Fig. 4e). *NRP1* encodes Neuropilin-1, a receptor in Semaphorin signaling pathway.^81,82^ *NRP1* showed concentrated expression in those cells, while *SEMA3A* were expressed mainly in those typical LGE MSNs co-expressing *EBF1* (Fig. 4e). Therefore, we assumed that this group of cells were possibly derived from MGE, and migrated passing along developing striatum to the cortex under the regulation of SEMA3 signaling pathway.^14^ CellChat analysis,^83^ a tool that can calculate cell–cell signaling pathways between clusters by predicting major signaling inputs and outputs, showed that SEMA3 signaling pathway was highly activated in the LGE group. In this pathway, *SEMA3A-*expressing cells might release the signal and *NRP1-*expressing cells would response to SEMA3A (Fig. 4f). Another highly expressed gene in those cells, *PEG10*, drew our attention. *PEG10* can produce long noncoding RNA (lncRNA) as well as PEG10 protein, both of which were reported to promoted cell migration in cancer cells.^84,85^ Previous study has shown that *MIR7-3HG* acted as a competing endogenous RNA to release *PEG10* RNA from microRNA27-A-3p.^86^ *MIR7-3HG* was also expressed at high levels in those cells from human samples (Supplementary information, Fig. S4c), which might regulate the *PEG10* expression. In addition, we downloaded the in situ sequencing data provided by Sun’s group^80^ and transformed signals into images. A group of *NKX2-1*^*+*^ and *LHX8*^+^ cells were located away from MGE at human GW12 (Supplementary information, Fig. S4d). These results indicate that a group of *LHX8*^+^ interneurons from MGE would migrate along LGE and be regulated by the SEMA3A-NRP1 signaling pathway, as well as the MIR7-3HG-PEG10 pathways during GE development (Fig. 4g). Combined with the pseudotime analysis, our researches on LGE revealed the regional genetic regulatory model (Fig. 4h).

### Transcriptional regulatory network in the human GE

**Fig. 5:**
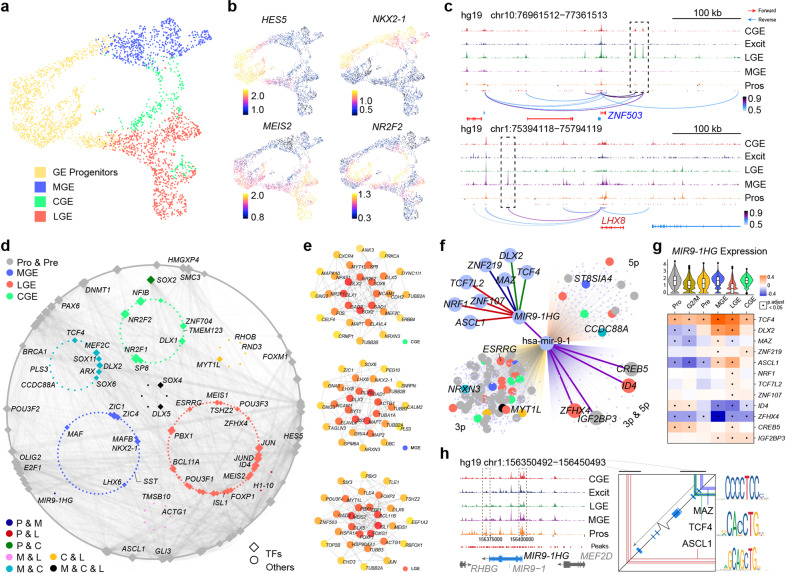
scATAC-seq analysis and heterogeneity of regulation between MGE and LGE. **a** Cell types in scATAC-seq visualized by UMAP after lift-over based on scRNA-seq data. **b** Accessible degrees of classic marker genes expressed in progenitors and different GE regions. **c** Differentially accessible chromatin peaks upstream of *ZNF503* (top) and *LHX8* (bottom). **d** Gene regulatory network generated based on integration of scRNA-seq and scATAC-seq. **e** Hub genes of different GE regions deduced by protein–protein interactions. **f** Predicted regulatory pathway of *MIR9-1HG*. Upstream of *MIR9-1HG* were divided into three groups based on predicted binding site represented by different line colors. Dot colors share legends as in **d**. **g** Expression pattern of *MIR9-1HG* in human integrated data and its correlation with other genes in different cell clusters. False discovery rates were adjusted by BH method to generate “P. adjust”. **h** Open region of *MIR9-1HG* and predicted related TF binding sites. Specific peaks in MGE or progenitor clusters compared with LGE were marked (left) and predicted binding sites of upstream TFs were mapped to these regions (right).

We also performed scATAC-seq of human GE at GW9 to investigate in more detail the transcription regulatory networks in human developing GEs. The single-cell assay from two technical replicates of scATAC-seq data was employed and we obtained a consensus set of 240,672 accessible peaks representing potential *cis*-regulatory elements (Supplementary information, Fig. S5a). We integrated the human scATAC-seq data with our GW9 scRNA-seq data by ArchR^87^ and found four major cell types matched between two sets of data (Fig. 5a and Supplementary information, Fig. S5b). We used the gene score, which is a prediction tool of gene expression based on the accessibility of regulatory elements, to visualize typical marker genes expression levels for GE progenitors, MGE, LGE and CGE on the UMAP plots (Fig. 5b). We built a marker gene list for the four major cell types based on the human scRNA-seq data (Supplementary information, Table. S2). *ZNF503* and *LHX8* were the representative makers of LGE and MGE lineages in the marker list (Supplementary information, Table S2 and Fig. S5c). We found two specific accessible genomic regions (open chromatin peaks) upstream of *ZNF503* in LGE and one unique peak upstream of *LHX8* in MGE (Fig. 5c). This unique peak of *LHX8* contains a highly H3K4Me3 modification region based on UCSF brain methylation database in UCSC browser (http://genome.ucsc.edu), as well as a predicted binding site for PRX2. Both peaks related to *ZNF503* contained predicted target motif of MEIS1, a marker of LGE^80^ (Supplementary information, Fig. S5d). These findings suggest the potential regulatory mechanisms of *LHX8* expression in MGE and *ZNF503* expression in LGE.

We then generated gene regulatory networks (GRNs) by linking distal *cis*-regulatory elements to individual genes using a method developed by Tu lab (see Method and Material) in order to identify candidate transcriptional factors (TFs) and regulators. Among all the regulatory pairs captured, we selected those upstream TFs and downstream genes that were both in the marker list visualized by Cytoscape^88^ (Fig. 5d). Each group of cells showed their specific as well as shared regulatory networks. For example, apart from well-known TFs such as NKX2-1 and LHX6, MGE also shared ARX and MEF2C with CGE, while NR2F2 and NFIB specifically regulated CGE development. LGE seemed to have a relative independent regulatory network mainly consisted of ISL1, FOXP1 and ZFHX4 (Fig. 5d). We got similar results by extracting the hub genes from three GE subregions based on protein–protein interaction (PPI) (Fig. 5e).

We next sought to search for unknown genes that might play important roles during GE development. *MIR9-1HG* (also named as *C1orf61*), the microRNA9-1 host gene, stood out from our results. It was involved in progenitors and MGE development (Fig. 5d). The high expression level of *MIR9-1HG* in progenitors and MGE clusters showed by feature plot confirmed our regulatory network results (Supplementary information, Fig. S5e). We looked through the potential upstream genes of *MIR9-1HG* in order to figure out its regulatory mechanism. Combined with the opening chromatin peaks around *MIR9-1HG* and potential binding motifs of TFs, three TFs, *MAZ, TCF4* and *ASCL1*, were selected from 8 candidates (Fig. 5f). MAZ has been reported as a transcriptional activator binding protein,^89^ suggesting that MAZ would support the expression of *MIR9-1HG*. The binding motif of TCF4, a TF involved in the initiation of neural differentiation,^90,91^ was mapped to the open chromatin peaks around the transcription start site of *MIR9-1HG* (Fig. 5). Correlation analysis among gene expression levels in different cell types showed the significantly positive correlation between *TCF4* and *MIR9-1HG* in all kinds of cells in our data (Fig. 5g and Supplementary information, Fig. S5e). This suggests that *MIR9-1HG* expression was positively regulated by TCF4. Next, we found ASCL1 binding motifs located at the open chromatin peaks downstream of *MIR9-1HG*, especially in the progenitor cluster (Fig. 5h). *ASCL1* showed significant negative correlation with *MIR9-1HG* in progenitors but positive correlation in MGE and LGE (Fig. 5g and Supplementary information, Fig. S5e). We assumed that ASCL1 might inhibit *MIR9-1HG* expression in the progenitors but not in MGE. We further inspect the potential downstream genes of *MIR9-1HG*. Since *MIR9-1HG* is a lncRNA and microRNA9-1 can be spliced or generated from it, we searched for the target genes of hsa-mir-9-1 from three different databases, TargetScanVert, miRDB and TargetMiner.^92–94^ Four genes were found to be regulated by both hsa-mir-9-1-3p and 5p in all those databases. Two of them were previously described as LGE specific TFs, ZFHX4 and ID4. The expression levels of these two genes showed remarkably negative correlation with *MIR9-1HG* in MGE and LGE (Fig. 5g). In MGE, *MIR9-1HG* was expressed at high levels where *ZFHX4* and *ID4* were barely expressed, while the gene expression patterns were opposite in LGE (Supplementary information, Fig. S5e). Altogether, these results propose a new regulatory pathway in which *MIR9-1HG* is regulated by ASCL1 and TCF4. *MIR9-1HG-*encoding hsa-mir-9-1 would inhibit the expression of *ZFHX4* and *ID4*. This may explain how the distinct fates of MGE and LGE are determined during early brain development in human.

## Discussion

In this study, we investigated the single-cell transcriptional identity of primate embryonic GEs and the gene regulatory networks that are potentially involved in cell fate determination in different subregions, as well as in migrating routes and destinations through scRNA-seq and scATAC-seq. Recently, a study adopted scRNA-seq and in situ sequencing to explore the origin of diverse interneurons in human fetal brains was published just before the submission of this manuscript. The study provided details on the temporal and spatial identity of interneuron precursors based on the expression of different genes.^80^ In addition, their in situ sequencing data will provide a rich resource for other groups including ours to confirm the expression of many genes. Another study comparing human and mouse interneuron development was published during the review of our article. The study also adopted scRNA-seq analysis to show details of the cell identities during human GE development as we did,^95^ but had not revealed the gene regulatory networks or unique developmental mechanism in human GEs compared to that in rodents. Focusing on the neurogenesis stage, we studied the early cell differentiation and migration in human and *Macaca fascicularis* developing MGE, LGE and CGE. Integration between human and macaque datasets revealed the well conserved GE cell diversities, the existence of oRGs in primate GEs and a novel regulatory pathway in human LGE. By combining the analyses of scATAC-seq and scRNA-seq data, we discovered the TFs that are potentially important for cell fate determination in different human GE regions, altogether with their gene regulatory networks including a novel regulatory pathway that may lead to the distinct fates of MGE and LGE.

oRGs are considered to be the major driving force for brain volume expansion in primates, especially human.^56^ Our results confirmed the existence of oRGs in human developing GE. This would explain the expansion and increased cell density of human GEs, compared with mouse GEs.^55,56,96^ Other genes found by differential gene expression analysis between human and mouse progenitors in GE partially matched the previous studies in cerebral cortex.^97^ For example, *FAM107A* is expressed in the oRGs of both cerebral cortex and GEs.^58^ In addition, we found that cell proliferation associated genes *TCIM* and *CLU* are expressed at high levels in the human GE progenitors.^58,59^ The comparison among macaque, human and mouse is hard to display. When we perform lift-over between the macaque and human genome, some human genes could not be found in the orthologous gene list downloaded from Ensembl V104 due to the incomplete annotation of the *Macaca fascicularis* genome. Nevertheless, we inspected those DEGs in macaque GE progenitors and found the conserved expression of *CLU* in primate RGCs, while *TCIM* was not expressed in macaque. The regulatory roles of those genes in the progenitors need further investigation.

The cortical interneurons mainly comprise PV^+^ or SST^+^ GABAergic interneurons derived from MGE, and VIP^+^ or RELN^+^ 5HT3aR^+^ interneurons derived from CGE.^5,18,71^ In mice, the early differentiation and molecular markers of GE interneurons have been well investigated.^17,37,39,71^ Our study has confirmed the cell diversity during MGE development but not CGE. Most cells in CGE expressed *NR2F2* and *NR2F1* but barely *VIP* and *RELN* (Supplementary information, Fig. S1h and Fig. S3f), the typical marker for CGE derived mature interneurons.^5,18^ We assume that more specific cell fates and functions in CGE remain unclear at the stages studied here. In MGE, we identified a small group of cells specifically expressing *CRABP1* and *ANGPT2*. Combined with the scRNA-seq analysis of PV^+^ interneurons and the developmental process of GE in mice,^17,98,99^ those cells were defined as the PV^+^ interneuron precursors derived from MGE.

The migration of GE interneurons begins at early embryonic stage and continues until the extinction of the germ layer in the brain. Several signal pathways have been shown to regulate the migration of interneurons. CXCL12/CXCR4 signaling controls the migration of interneurons during brain development.^68,69^ We detected the expression of *ERBB4* and *CXCR4* in the SST^+^ interneuron lineage, as well as *CXCL12* in the LGE lineage. Our results indicate that the migration of SST^+^ interneurons is under the regulation of CXCL12/CXCR4 signaling pathway, mainly through the CXCL12 released from LGE. The other cell migration related pathway is the SEMA3-NRP signaling, with SEMA3A repulsing the migration of NRP1^+^ neurons.^14,81,82,100,101^ In LGE, we detected the expression of *SEMA3A*, *NRP1* and *NRP2*. However, *SEMA3A* is expressed in the typical striatal interneurons, while *NRP1* is expressed in a group of MGE-derived interneurons. This is consistent with previous reports that striatal interneurons will inhibit cortical interneurons derived from MGE from straying into striatum through SEMA3-NRP signaling pathway in mouse.^14^ The in situ sequencing data provided by Sun’s group^80^ also proved that a group of *NKX2-1*^*+*^ and *LHX8*^*+*^ cells were located near LGE at human GW12 (Supplementary information, Fig. S4d). In this case, our results indicate that some *NKX2-1*^+^ and *LHX8*^+^ interneurons in primate MGE would pass along LGE towards their specific destination under the regulation of SEMA3A-NRP signaling pathway. Another potential regulatory network we found in the migrating MGE cells was the *MIR7-3HG*-*PEG10* pathway. *PEG10* were expressed both in those *LHX8*^+^ interneurons and some striatal interneurons. In human cancer cells, both lncRNA and proteins encoded by *PEG10* were verified to promote cell migration and invasion.^84,85^ Recently, a study demonstrated that PEG10 could package its own mRNA for secretion in both human and mouse.^102^ Altogether, these findings suggest that PEG10 promotes the interneuron migration and is involved in intercellular communication during human GE development. However, the annotations of lncRNA are not so precise in *Macaca fascicularis* genome compared with protein coding genes. That is why we could not identify the homologous gene of human *MIR7-3HG* in macaque. Our analysis of the developmental MGE and LGE revealed the regional genetic regulatory model and a new possible gene regulatory pathway influencing the surrounding cells.

During brain development, the differences in gene expression determine the diversity among brain regions. The regional characteristics are regulated by different and complicated gene sets, instead of one or two genes. The major regulatory networks of GEs have been identified previously. For instance, MGE development is considered to be induced by sonic Hedgehog (SHH) signaling pathway in the ventral forebrain. *NKX2-1* expression is induced by SHH. NKX2-1 then stimulates the expression of *LHX6*, and *SOX6* which is required for SST^+^ and PV^+^ interneurons development.^13,103^ scATAC-seq enabled us to find more potential regulatory networks during human GE development. We identified *MIR9-1HG* as a potential master regulatory gene in GE progenitors and MGE. ASCL1 and TCF4 regulate its expression, and the encoded *mir9-1* might suppress the expression of *ZFHX4* and *ID4* in MGE. However, the very upstream genes of this regulatory pathway remain to be disclosed. Our study uncovered several crucial TFs in different regions of human GE. Moreover, we found a possible specific pathway that participates in the regulation of differential gene expression between MGE and LGE.

In summary, our study demonstrates the strong conservation of cell diversity and lineage characteristics in GEs between human and *Macaca fascicularis*. We found some DEGs between human and mouse GE progenitors and discovered the presence of oRGs in primate GE. Moreover, our study verified the conservation of gene regulatory networks in the developing primate GE and explored the new mechanisms involved in cell migration and fate determination of different GE regions.

## Materials and methods

### Human sample collection

The human clinical tissues of pregnancies at GW 9 and 13 were obtained upon therapeutic termination of pregnancy at Peking University Third Hospital and department of obstetrics and gynecology at Peking Union Medical College Hospital, Beijing, China. An informed consent document was signed by the patient before the collection of the human sample. The whole experiment was examined by Ethics Committee of Peking University Third Hospital (M2021461) and Peking Union Medical College Hospital (JS-2471).

### *Macaca fascicularis* subjects

The macaque samples at GW7, 10, 12 were collected at Beijing Institute of Xieerxin Biology Resource under the supervision of veterinarian. All surgery programs were approved by the Institutional Animal Care and Use Committee of Xieerxin Biology Resource (XEX20212025).

### Brain tissue dissection and cells dissociation

The human and macaque brain tissues were dissected in ice-cold normal saline under the dissection microscope. GEs from all five samples were collected. Half of the human GW 9 GE was stored in liquid nitrogen for scATAC-seq. Half of the human GW13 brain was stored in 4% paraformaldehyde (PFA) for immunostaining. Each tissue for scRNA-seq was dissociated in 500 μL dissociation agent (400 U/mL DNaseI on hibernate E buffer, 10 U/mL papain) at 37°C on a thermocycler for 15 min. Dissociation was terminated by 500 μL 10% FBS in Hibernate E buffer. Cells were centrifuged at 4°C for 5–10 min. After removing the supernatant, 1 mL HA buffer was added in and cells were dispersed into single-cell suspension by repetitive pipetting.

### sc-RNA Seq

Using single cell 3′ GEM, Library & Gel Bead Kit V3.1 (10× Genomics, 1000075) and Chromium Single Cell B Chip Kit (10× Genomics, 1000074), the prepared cell suspension (300–600 living cells per μL determined by Count Star) was loaded onto the Chromium single cell controller (10× Genomics) to generate single-cell gel beads in the emulsion according to the manufacturer’s protocol. Then single cells were suspended in PBS containing 0.04% BSA. The target cell will be recovered to about 8000 cells by estimation. Captured cells were lysed to release their RNA, which were then barcoded through reverse transcription in individual GEMs. Then reverse transcription was performed on a S1000TM Touch Thermal Cycler (Bio Rad) at 53°C for 45 min, followed by 85°C for 5 min. The cDNA was kept at 4°C, then was amplified and the quality was assessed using an Agilent 4200 (performed by CapitalBio Technology, Beijing).

### Single-cell gene expression quantification

Cell Ranger (v5.0.1) was used to convert raw sequencing data to FastQ format, perform quality control and read counting of gene with default parameters. Human samples were mapped to the GRCh38 (hg38) and Macaque samples were mapped to the (v5.0). Gene-cell data matrix was analyzed by Seurat (v4.0.4), and we used MiQC (v1.1.3)^104^ (posterior.cutoff = 0.95, model.slot = “flexmix_model”, model.type = “spline”) to remove low quality genes. Cells expressing more than 20% of mitochondrial genes were also excluded. We also removed the cells expressing less than 800 genes or more than 6000 genes. Cells that expressed hemoglobin genes were also filtered out. Then we performed doublet analysis using DoubletFinder (v.2.0.3)^105^ and removed the predicted doublet. Count data were normalized and scaled by the SCTransform function.

### Identification of marker genes among clusters in scRNA-seq and cell type annotation

Principal Component 1–11 were used to cluster the cells by FindClusters function under the resolution of 0.9. The marker genes of each cluster were identified by using the FindAllMarkers function (thresh.use = 0.25, only.pos = TRUE, min.pct = 0.25, logfc.threshold = 0.5, test.use = “wilcox”). Genes with *P* < 0.05 were selected as markers. Clusters were visualized by UMAP and verified by several well-known marker genes. RGC and IPCs of GE showed high expression level of *HES5* or *ASCL1*. MGE comprised cells specifically expressing *NKX2-1 *and *LHX6*, while *MEIS2*, *ISL1* in LGE and *CALB2*, *NR2F2* in CGE, microglia and pericytes could be discerned by their corresponding markers, namely *CX3CR1* and *COL3A1*. And we used FindMarkers function (thresh.use = 0.5, only.pos = TRUE, min.pct = 0.25, logfc.threshold = 0.5, test.use = “wilcox”) to find specific genes in each individual cluster.

### Integrate human and *Macaca fascicularis* scRNA-seq by seurat3.0

Seurat3.0^45^ was used to integrate different assay based on species by the function FindIntegrationAnchors and IntegrateData with default parameters. Then we used RunPCA function (reduction = “pca”, npcs = 50) to get the Principal Component of the assay, RunUMAP function (reduction = “pca”, dims = 1:11) to visualize data, FindNeighbors function (dims = 1:11) and FindClusters function (resolution = 0.9) to get biological significant clusters. And we performed Glmnet (v4.1-3) to calculate the expression similarity between different species via penalized maximum likelihood (alpha = 0.99, nfolds = 10), we also compared the cluster annotation based on integrated assays with individual assays and visualized it in Sankey plot.

### Integrate human scRNA-seq by seurat3.0

The method was just similar to previously mentioned and we integrated matrix based on different time points. As to further cluster and analysis the important clusters, the same pipeline was used. We used RunPCA, FindNeighbors, FindClusters, RunUMAP, FindAllMarkers and FindMarkers functions on four main cell types (GE progenitors, MGE, CGE, LGE). The PC number we used was 18 and the resolution we used was 0.65.

### Integrate mouse scRNA-seq by seurat3.0

All the GEO datasets were obtained from the GEO website (GEO: GSE142768, GSE184879), which is publicly accessible at https://www.ncbi.nlm.nih.gov/geo. SCTransform function was used to normalize and scale the count matrix. Two datasets of E12.5 mouse MGE and LGE cells were integrated and calculated by the same Seurat pipeline. We performed FindIntegrationAnchors and IntegrateData function with default parameters to integrate two Seurat objects. Then we used 16 principal Components calculated by RunPCA function to construct the Shared Nearest Neighbor graph by FindNeighbors function. FindClusters function was performed to get the clusters (resolution = 0.5). Three MGE data of E11, E14 and E17 used the same Seurat pipeline (dims = 1:16, resolution = 0.5).

### Cell-cycle analysis

We used Seurat package-supplied cell cycle related genes, 43 genes of S phase and 54 genes of G2/M phase, to calculate the stage of the cells by performing the CellCycleScoring function in Seurat.

### Gene Ontology (GO) enrichment analysis

We used clusterProfiler (v4.1.4)^106^ to get enriched GO terms of marker genes. We picked genes (*P* < 0.05) found in previous study and used enrichGO function (ont = “BP”, pAjustMethod = “BH”, pvalueCutoff = 0.05, qvalueCutoff = 0.05).

### Trajectory analysis

Splicing-specific count data were calculated by Velocyto (v.0.17.17)^52^ for downstream RNA velocity analysis with default parameters. The resulting loom files were then further analyzed using the scVelo (v0.2.3).^53^ We performed gene selection, normalization (min_shared_counts = 30, n_top_genes = 2000), moment estimation (n_pcs = 30, n_neighbors = 30), and estimation of RNA velocities. We used the stream embedding function to visualize RNA velocities on UMAP. Monocle 3^70^ was also used to infer the pseudotime trajectories of the major clusters. Monocle “cds” was constructed by our data calculated by Seurat. Then we defined the cells showed as precursor as the “root_state”.

### Cell–cell communication analysis

Cell**–**cell interactions between each cluster were identified and visualized by CellChat (v1.1.2).^83^ Seurat objects for human sample were merged, createCellChat function was applied to create a CellChat object from Seurat normalized data, then addMeta and setIdent function were used to add clusters labeled in previous Seurat object. We focused on the CellChat human database and performed “identifyOverExpressedGenes”, “identifyOverExpressedInteractions” functions to identify over-expressed ligands or receptors. Then we used “projectData” function to project gene expression data onto PPI network. “computeCommunProb” and “filterCommunication” functions (min. cells = 10) were used to compute communication probability and infer cellular communication network. “computeCommunProbPathway” and “aggregateNet” functions were performed to infer the cell–cell communication at a signaling pathway level between each cluster.

### Nuclei isolation from frozen brain tissue

Nuclei from previous frozen GEs of human GW9 were isolated. Tissue was cut into small pieces and ground in 2 mL of ice-cold homogenization buffer [20 mM Tris, pH 8.0 (Thermo Fisher Scientific)], 500 mM sucrose (Sigma), 50 mM KCl (Thermo Fisher Scientific), 10 mM MgCl_2_ (Thermo Fisher Scientific), 0.1% NP-40 (Roche), 1× protease inhibitor cocktail (Roche), and 1% nuclease-free BSA, and 0.1 mM (DTT). To release the nuclei, tissues were homogenized by strokes. 30 μM cell strainer was used to filter nuclei into a 15 mL centrifuge tube. After centrifuging for 5 min at 4°C, nuclei were obtained and washed twice with 1 mL of ice-cold blocking buffer (1× PBS supplemented with 1% BSA). Another centrifuging was performed and the nuclei were collected in 50 μL of 1× PBS containing 1% BSA and counted by DAPI staining.

### scATAC-Seq library preparation and sequencing

We used DNBelab C Series Single-Cell ATAC Library Prep Set (MGI, #1000021878^107^) to prepare single-cell ATAC-seq libraries. We then created the human GE library. The transposed single-nucleus suspensions were converted to barcoded scATAC-seq libraries. After procedures including droplet encapsulation, pre-amplification, emulsion breakage, capture beads collection, DNA amplification, and purification, indexed sequencing libraries were prepared according to the user guide. We measured the concentrations of sequencing library with Qubit ssDNA Assay Kit (Thermo Fisher Scientific). The library was sequenced using a paired-end 50 sequencing scheme by the BGISEQ-500 platform at China National GeneBank (CNGB).^108^

### scATAC-seq process

Raw sequencing reads from BGISEQ-500 sequencer were filtered and demultiplexed using PISA, and aligned to the hg19 human genome. Fragment data were further processed using ArchR (V2.0.1).^87^ The cells with transcription start site (TSS) enrichment score less than five and fragment number less than 1000 were removed. We did doublet analysis by “addDoubletScores” and “filterDoublets” functions in AchrR. Then we performed “iterative LSI” and used major pc2-pc30 to cluster by Seurat’s “FindClusters” function with resolution 0.6. We used gene activity scores to identified different cell types for various marker genes. Clusters were verified by several well-known marker genes as previous described. 240672 peaks were found by performed Peak calling using MACS2 (v2.1.1), and we can link peak to gene by the addPeak2GeneLinks function. We can also use CIS-BP database to identify accessible *cis*-regulatory elements (CREs).

### Identification of marker genes among clusters in scATAC-seq

We use “getMarkerFeatures” function (useMatrix = “GeneScoreMatrix”, bias = c (“TSSEnrichment”, “log10 (nFrags)”, testMethod = “wilcoxon”)) and FDR ≤ 0.01 & |Log2FC| ≥ 1 to find marker genes among clusters in scATAC-seq.

### Build gene regulatory networks

To match single-cell transcriptomes and epigenomes, we combined scATAC-seq with human GW9 scRNA-seq. We performed “addGeneIntegrationMatrix” function to integrate gene expressing matrix of scRNA-seq on “geneScoreMatrix” of scATAC-seq. Then we predicted *cis*-linkages between CREs and downstream genes. Moreover, we inferred *cis*-regulatory interactions and defined a TF-gene GRN. This method made by Tu lab has not been published. We extracted important TFs based on the marker genes found in major clusters (GE progenitors, MGE, CGE, LGE). The hub gene was calculated based on PPI within the genes found in GRN. And networks were visualized in Cytoscape (v3.9.0).

### Immunofluorescence

Brains were fixed in 4% PFA before treatment and dehydrated in 30% sucrose for cryosections. Tissue sections of 20 μm sectioned by Leica CM 1950 were used for further immunostaining. Sections were processed by blocking buffer (3% BSA + 10% FBS + 0.3% Triton X-100 in 1× PBS) for 1 h, and incubated by primary antibodies of HOPX (Sigma, HPA030180) at 4°C overnight. After washing by 1× PBS three times for 10 min, sections were incubated by secondary antibodies and DAPI for 1 h. Immunofluorescence pictures were taken using Zeiss confocal LSM 800.

## Supplementary information

Fig. S1

Fig. S2

Fig.S3

Fig.S4

Fig. S5

Fig. S6

Table S1

Table S2

## Data Availability

The single cell sequencing data were uploaded to China National Center for Bioinformation (https://www.cncb.ac.cn/) with human data in PRJCA007893 and *Macaca fascicularis* (crab-eating monkey) data in PRJCA007270. The scATAC-seq data reported in this study are also available in the CNGB Nucleotide Sequence Archive (CNSA: https://db.cngb.org/cnsa; accession number CNP0002651).
