# Author Correction: Evolutionarily conservative and non-conservative regulatory networks during primate interneuron development revealed by single-cell RNA and ATAC sequencing

**DOI:** 10.1038/s41422-022-00757-0

**Published:** 2022-12-16

**Authors:** Ziqi Zhao, Dan Zhang, Fuqiang Yang, Mingrui Xu, Shaoli Zhao, Taotao Pan, Chuanyu Liu, Yongjie Liu, Qingfeng Wu, Qiang Tu, Ping Zhou, Rong Li, Jia Kang, Lan Zhu, Fei Gao, Yaqing Wang, Zhiheng Xu

**Affiliations:** 1 State Key Laboratory of Molecular Developmental Biology, CAS Center for Excellence in Brain Science and Intelligence Technology, School of Future Technology, Institute of Genetics and Developmental Biology, Chinese Academy of Sciences, Beijing, China; 2 University of Chinese Academy of Sciences, Beijing, China; 3 BGI-Beijing, Beijing, China; 4 BGI-Shenzhen, Shenzhen, China; 5 State Key Laboratory of Molecular Developmental Biology, Institute of Genetics and Developmental Biology, Innovation Academy for Seed Design, Chinese Academy of Sciences, Beijing, China; 6 Key Laboratory of Genetic Network Biology, Institute of Genetics and Developmental Biology, Chinese Academy of Sciences, Beijing, China; 7 Center for Reproductive Medicine, Department of Obstetrics and Gynecology, Peking University Third Hospital, Beijing, China; 8 Department of Obstetrics and Gynecology, Peking Union Medical College Hospital, Beijing, China; 9 State Key Laboratory of Reproductive Biology, Institute of Zoology, Chinese Academy of Sciences, Beijing, China

**Keywords:** Transdifferentiation, Genome-wide association studies

Correction to: *Cell Research* 10.1038/s41422-022-00635-9, published online 10 March 2022

We found that the information in the original Supplementary Figs. S2–S4 was not fully displayed due to an error in the image upload. The correct images are shown below for better display. These corrections do not affect the description of the results or the conclusion of this work. No change of the original figure legends is needed. We apologize for the mistake. The original article has been corrected.


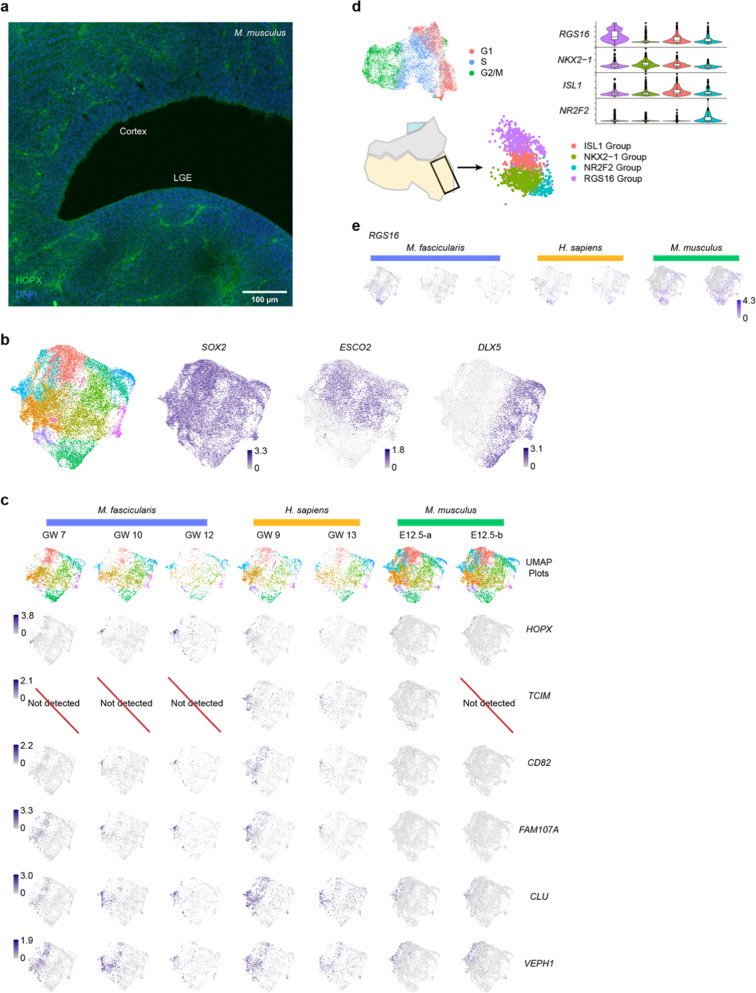


**Fig. S2** Transcriptional features of progenitors in different species


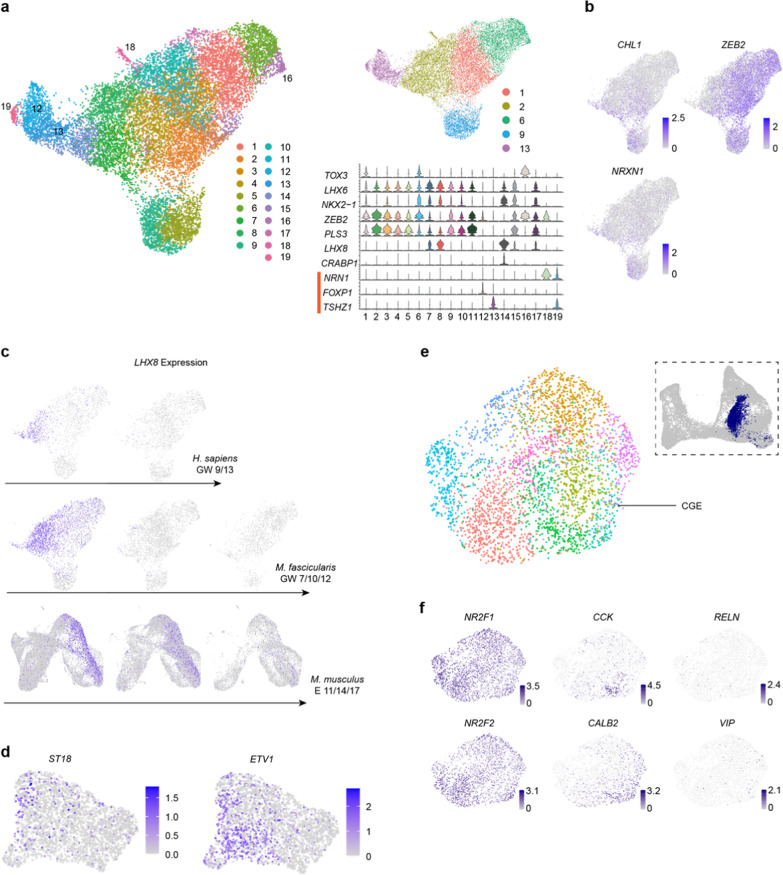


**Fig. S3** Extended data related to MGE and CGE development


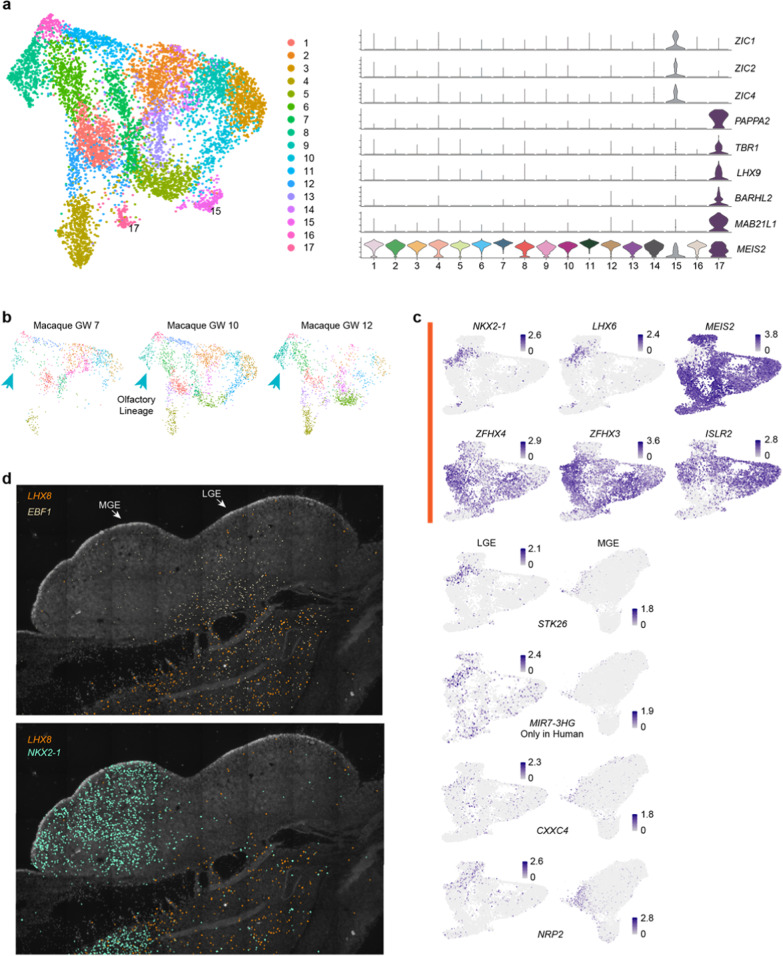


**Fig. S4** Different gene expression in primate developing LGE

