# Supplementary material for: Evolutionarily conservative and non-conservative regulatory networks during primate interneuron development revealed by single-cell RNA and ATAC sequencing
Source: Cell Res. 2022 Mar 10;32(5):425–36. doi: 10.1038/s41422-022-00635-9 (PMC9061815; doi:10.1038/s41422-022-00635-9)
Supplement: Supplementary file 1 — Fig. S1 [file 41422_2022_635_MOESM1_ESM.pdf]

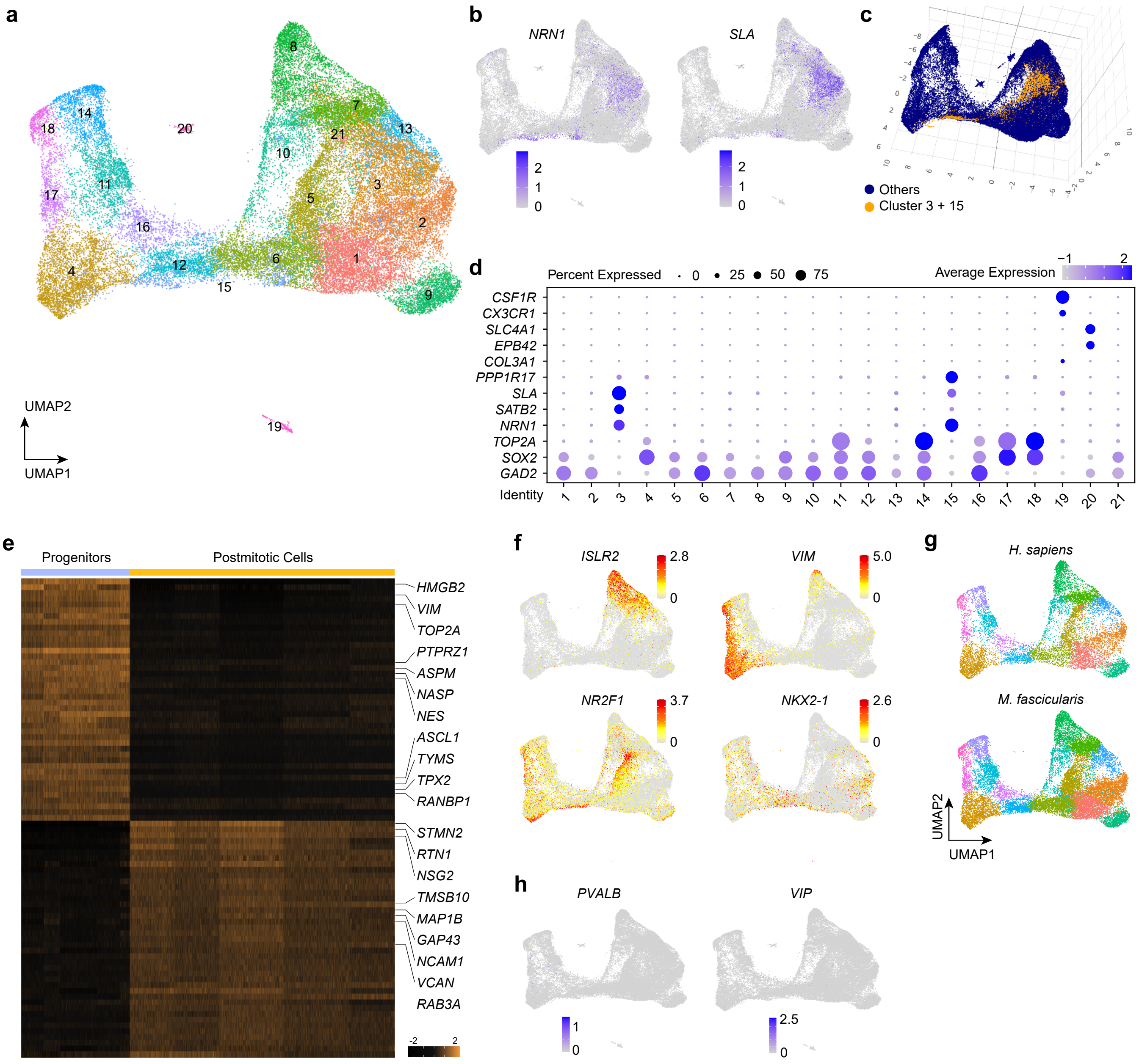

a
UMAP2
UMAP1
b
NRN1
SLA
c
Others
Cluster 3 + 15
d
Percent Expressed
Average Expression
CSF1R
CX3CR1
SLC4A1
EPB42
COL3A1
PPP1R17
SLA
SATB2
NRN1
TOP2A
SOX2
GAD2
Identity
e
Progenitors
Postmitotic Cells
HMGB2
VIM
TOP2A
PTPRZ1
ASPM
NASP
NES
ASCL1
TYMS
TPX2
RANBP1
STMN2
RTN1
NSG2
TMSB10
MAP1B
GAP43
NCAM1
VCAN
RAB3A
f
ISLR2
VIM
NR2F1
NKX2-1
g
H. sapiens
M. fascicularis
UMAP2
UMAP1
h
PVALB
VIP

**Fig. S1. Integration data of primate GEs**

**a.** Unsupervised cell clusters after quality control and integration.

**b.** Expression pattern of well-known markers of excitatory neurons. Both of them were relatively specifically expressed in cluster 3 and 15.

**c.** UMAP visualization in 3d form showing continuance between cluster 3 and 15.

**d.** Dot plot illustrating expression of marker genes in excitatory neurons, blood cells and microglia.

**e.** Heatmap showing the differentially expressed genes in GE progenitors and post-mitotic cells in primate GEs.

**f.** Additional markers of different cell types.

**g.** Cell distribution from human and macaque samples.

**h.** Expression levels of *PVALB* and *VIP* in our data presented by UMAP.
