# Supplementary material for: Evolutionarily conservative and non-conservative regulatory networks during primate interneuron development revealed by single-cell RNA and ATAC sequencing
Source: Cell Res. 2022 Mar 10;32(5):425–36. doi: 10.1038/s41422-022-00635-9 (PMC9061815; doi:10.1038/s41422-022-00635-9)
Supplement: Supplementary file 2 — Fig. S2 [file 41422_2022_635_MOESM2_ESM.pdf]

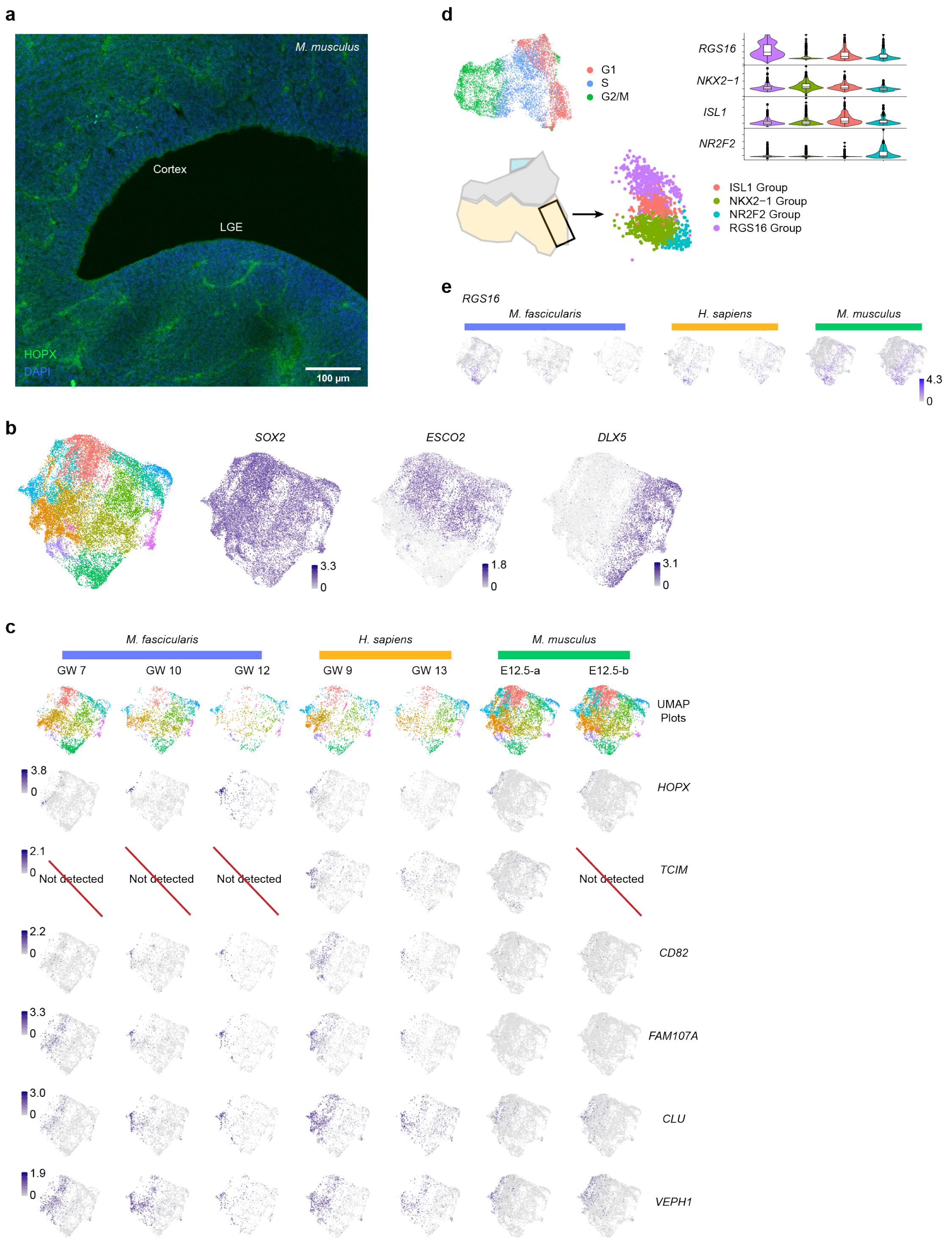

a
M. musculus
Cortex
LGE
HOPX
DAPI
100 μm
d
G1
S
G2/M
RGS16
NKX2−1
ISL1
NR2F2
ISL1 Group
NKX2−1 Group
NR2F2 Group
RGS16 Group
e
RGS16
M. fascicularis
H. sapiens
M. musculus
4.3
0
b
SOX2
ESCO2
DLX5
3.3
0
1.8
0
3.1
0
c
M. fascicularis
H. sapiens
M. musculus
GW 7
GW 10
GW 12
GW 9
GW 13
E12.5-a
E12.5-b
UMAP Plots
3.8
0
HOPX
2.1
0
Not detected
Not detected
Not detected
Not detected
TCIM
2.2
0
CD82
3.3
0
FAM107A
3.0
0
CLU
1.9
0
VEPH1

Fig. S2. Transcriptional features of progenitors in different species

a. Immunostaining of HOPX in mouse GE and cortex. DAPI stained for nuclei.

b. UMAP visualization of primate and mouse GE progenitors. Expression of *SOX2*, *ESCO2* and *DLX5* representing progenitors, cells in G2 phase and IPCs, respectively.

c. Comparison of expression profiles of glia cells from human, macaque and mouse. Cell distribution from different development stage of each species visualized in UMAP (top). Expression of some differentially expressed genes mentioned at **Fig. 2e** in seven samples were listed (bottom).

d. Expression levels of *RGS16* in IPCs. Cell cycle stages were recognized by Seurat 3 (top). Four new groups were generated by unsupervised clustering of two IPCs clusters in the integrated data (middle) and they were named based on their gene expression pattern (bottom).

e. Expression levels of *RGS16* in progenitors in three species visualized by UMAP.
