# Supplementary material for: Evolutionarily conservative and non-conservative regulatory networks during primate interneuron development revealed by single-cell RNA and ATAC sequencing
Source: Cell Res. 2022 Mar 10;32(5):425–36. doi: 10.1038/s41422-022-00635-9 (PMC9061815; doi:10.1038/s41422-022-00635-9)
Supplement: Supplementary file 3 — Fig.S3 [file 41422_2022_635_MOESM3_ESM.pdf]

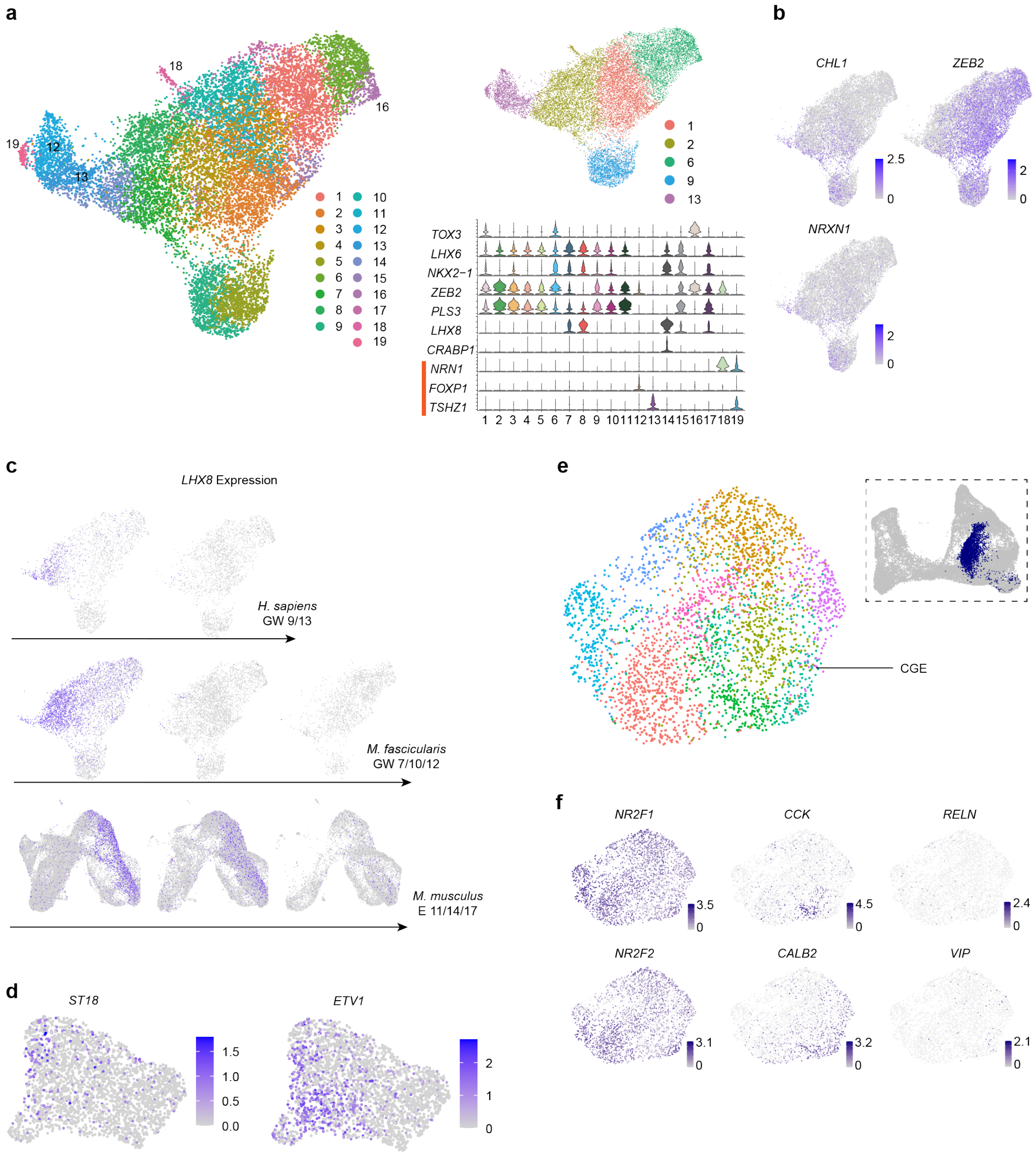

a
b
CHL1
ZEB2
NRXN1
TOX3
LHX6
NKX2−1
ZEB2
PLS3
LHX8
CRABP1
NRN1
FOXP1
TSHZ1
c
LHX8 Expression
H. sapiens
GW 9/13
M. fascicularis
GW 7/10/12
M. musculus
E 11/14/17
d
ST18
ETV1
e
CGE
f
NR2F1
CCK
RELN
NR2F2
CALB2
VIP

Fig. S3. Extended data related to MGE and CGE development

a. Clusters generated by unsupervised clustering method (left). Five MGE related clusters from integrated data were selected (top right) and non-MGE-derived cells were filtered finally based on genes labeled with orange bar in violin plot (bottom right).

b. Expression levels of axon related genes in mature lineages (left). Profile of gene co-expressed in *SST*+ lineage was also depicted (right).

c. Faded expression of *LHX8* in MGE along with embryonic development in human, macaque and mouse.

d. UMAP visualization of *ETV1* and *ST18* co-expressed with *CRABP1*.

e. Cell clusters from CGE were selected (top right) and further characterized (left, visualized by UMAP).

f. Expression levels of typical CGE derived interneurons markers visualized by UMAP.
