# Supplementary material for: Evolutionarily conservative and non-conservative regulatory networks during primate interneuron development revealed by single-cell RNA and ATAC sequencing
Source: Cell Res. 2022 Mar 10;32(5):425–36. doi: 10.1038/s41422-022-00635-9 (PMC9061815; doi:10.1038/s41422-022-00635-9)
Supplement: Supplementary file 4 — Fig.S4 [file 41422_2022_635_MOESM4_ESM.pdf]

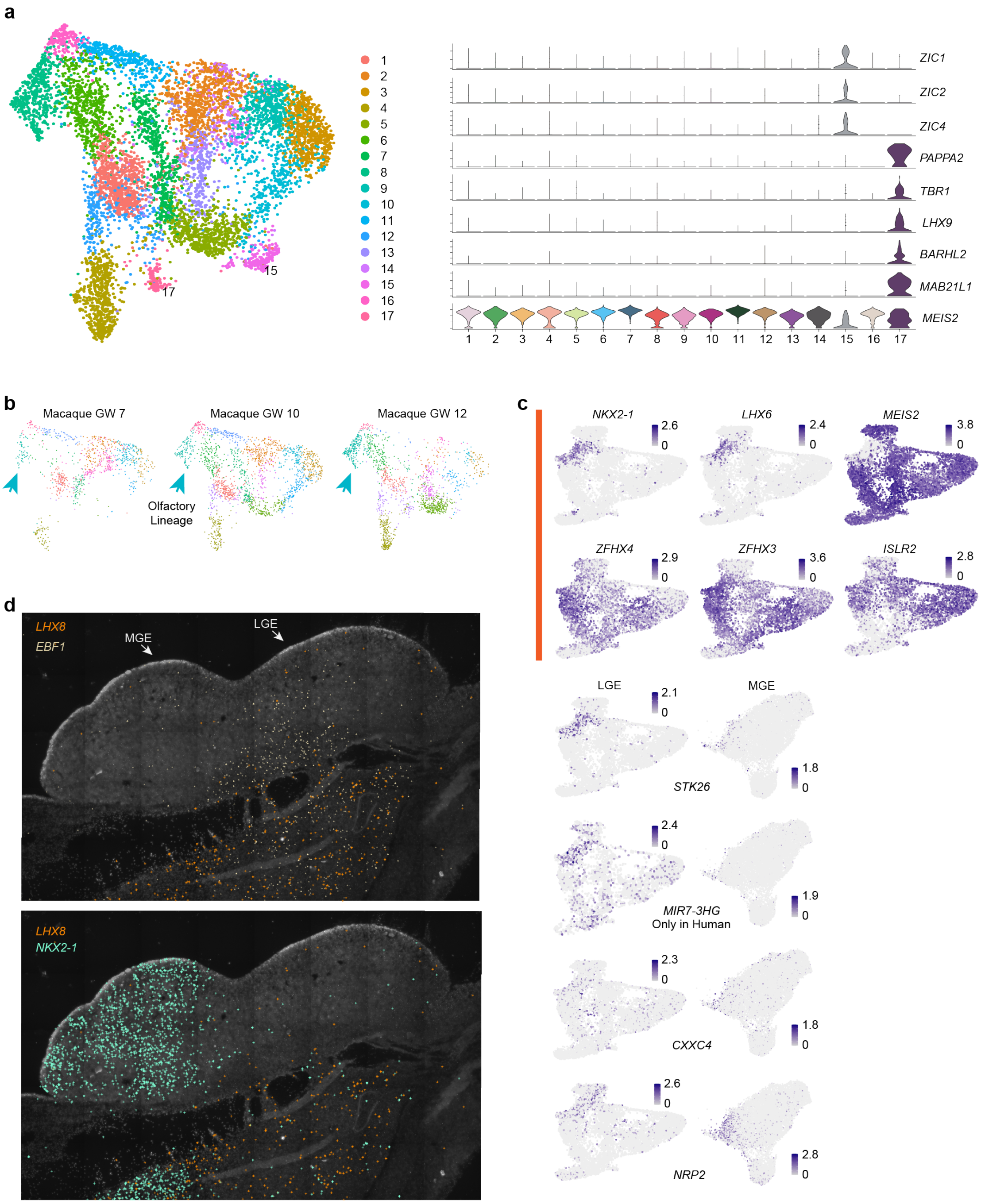

a
ZIC1
ZIC2
ZIC4
PAPPA2
TBR1
LHX9
BARHL2
MAB21L1
MEIS2
b
Macaque GW 7
Macaque GW 10
Macaque GW 12
Olfactory Lineage
c
NKX2-1
LHX6
MEIS2
ZFHX4
ZFHX3
ISLR2
LGE
MGE
STK26
MIR7-3HG
Only in Human
CXXC4
NRP2
d
LHX8
EBF1
MGE
LGE
LHX8
NKX2-1

Fig. S4. Different gene expression in primate developing LGE

a. Unsupervised LGE cell clusters visualized by UMAP (left). Cluster 14 and 16 were removed due to their representative makers of septum and excitatory neurons (right).

b. Cell distribution of three macaque LGE data in UMAP split by time. Cyan arrow marked olfactory lineages in different samples.

c. Expression levels of some typical LGE markers and MGE markers in LGE *LHX8*+ cells labeled by orange bar. Characteristics of specific expressed genes in LGE *LHX8*+ cells in both LGE and MGE.

d. Signals in the *in situ* sequencing data of GW 12 from Sun's group were transformed into images which illustrate the existence of *LHX8*+ lineages nearby the LGE.
