# Supplementary material for: Evolutionarily conservative and non-conservative regulatory networks during primate interneuron development revealed by single-cell RNA and ATAC sequencing
Source: Cell Res. 2022 Mar 10;32(5):425–36. doi: 10.1038/s41422-022-00635-9 (PMC9061815; doi:10.1038/s41422-022-00635-9)
Supplement: Supplementary file 5 — Fig. S5 [file 41422_2022_635_MOESM5_ESM.pdf]

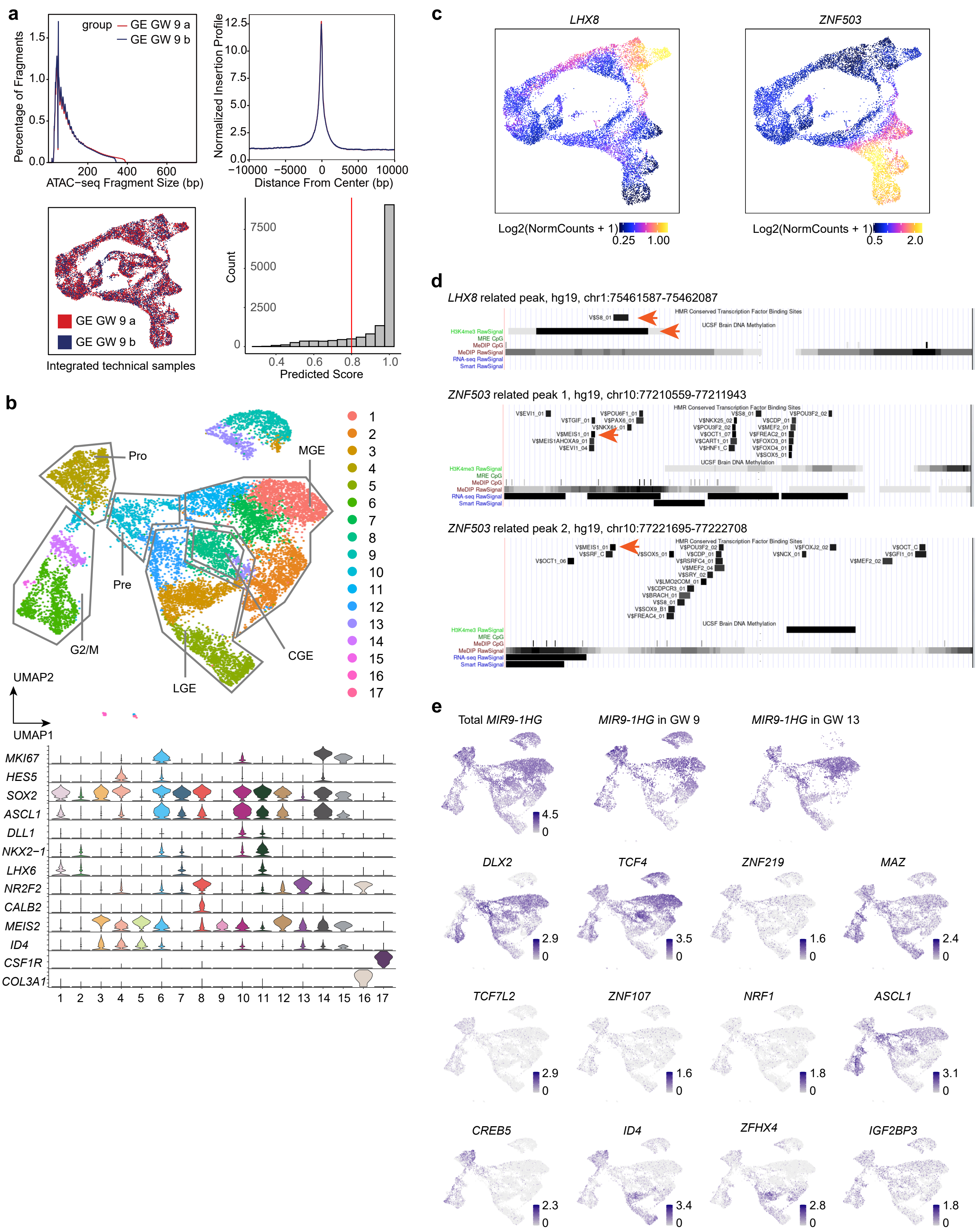

a
group
GE GW 9 a
GE GW 9 b
Percentage of Fragments
ATAC−seq Fragment Size (bp)
Normalized Insertion Profile
Distance From Center (bp)
GE GW 9 a
GE GW 9 b
Integrated technical samples
Count
Predicted Score
b
Pro
MGE
Pre
G2/M
LGE
CGE
UMAP2
UMAP1
MKI67
HES5
SOX2
ASCL1
DLL1
NKX2−1
LHX6
NR2F2
CALB2
MEIS2
ID4
CSF1R
COL3A1
c
LHX8
ZNF503
Log2(NormCounts + 1)
Log2(NormCounts + 1)
d
LHX8 related peak, hg19, chr1:75461587-75462087
ZNF503 related peak 1, hg19, chr10:77210559-77211943
ZNF503 related peak 2, hg19, chr10:77221695-77222708
HMR Conserved Transcription Factor Binding Sites
UCSF Brain DNA Methylation
H3K4me3 RawSignal
MRE CpG
MeDIP CpG
MeDIP RawSignal
RNA-seq RawSignal
Smart RawSignal
e
Total MIR9-1HG
MIR9-1HG in GW 9
MIR9-1HG in GW 13
DLX2
TCF4
ZNF219
MAZ
TCF7L2
ZNF107
NRF1
ASCL1
CREB5
ID4
ZFHX4
IGF2BP3

Fig. S5. scATAC-seq analysis of human GW9 GE

a. Fragment size distributions for the cells (top left) and aggregate TSS insertion profiles centered at all TSS regions (top right) for each sample in the GW9 GE scATAC-seq dataset. Line color represented two technical replicate samples from the dataset.

UMAP of integrated GW9 GE scATAC-seq dataset from two technical replicates (bottom left). Bar plot of cells predicted scores representing the similarity of scATAC-seq and scRNA-seq data calculated by ArchR pipeline. Vertical red line marked the accessible predicted scores 0.8 (bottom right).

b. Unsupervised cell clusters and cell types of integrated human GW9 and GW13 GEs on UMAP (Top). Violin plot of representative markers of GE clusters (Bottom).

c. Accessible degrees of *LHX8* and *ZNF503* visualized via UMAP. Same patterns were observed between these genes and corresponding GE marker genes.

d. Views of open chromatin peaks related to *LHX8* and *ZNF503* in UCSC genome browser. Conserved TFBS and UCSF brain methylation database were addressed. S8 also named PRX2. Location of methylation site and TF binding motifs were marked with orange arrows.

e. *MIR9-1HG* expression in GEs visualized based on UMAP (top). Gene of predicted upstream TFs of *MIR9-1HG* and downstream of mir9-1 expression in GEs (bottom).
