# Supplementary material for: Evolutionarily conservative and non-conservative regulatory networks during primate interneuron development revealed by single-cell RNA and ATAC sequencing
Source: Cell Res. 2022 Mar 10;32(5):425–36. doi: 10.1038/s41422-022-00635-9 (PMC9061815; doi:10.1038/s41422-022-00635-9)
Supplement: Supplementary file 6 — Fig. S6 [file 41422_2022_635_MOESM6_ESM.pdf]

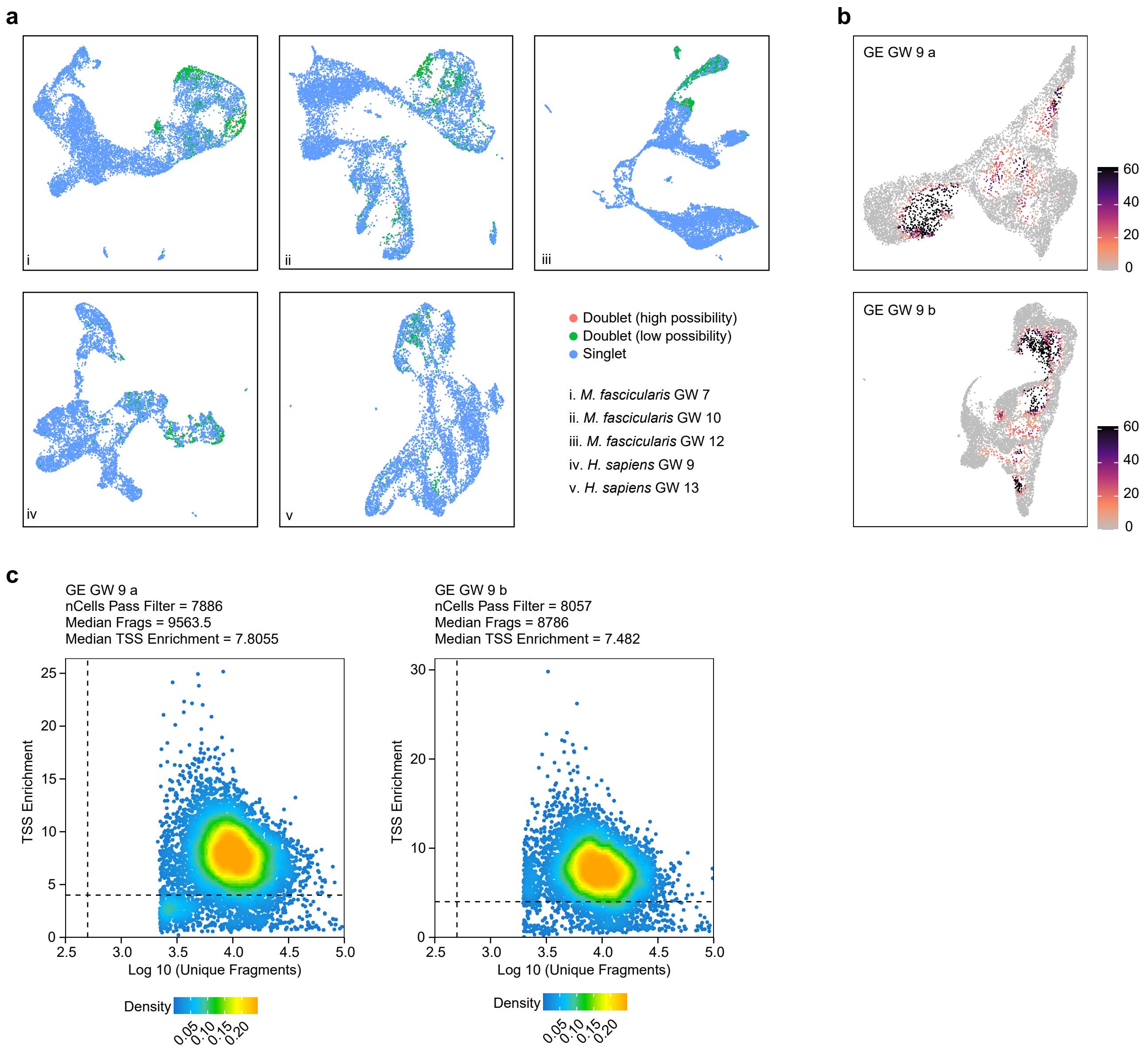

a
b
i
ii
iii
iv
v
GE GW 9 a
GE GW 9 b
60
40
20
0
Doublet (high possibility)
Doublet (low possibility)
Singlet
i. M. fascicularis GW 7
ii. M. fascicularis GW 10
iii. M. fascicularis GW 12
iv. H. sapiens GW 9
v. H. sapiens GW 13
c
GE GW 9 a
nCells Pass Filter = 7886
Median Frags = 9563.5
Median TSS Enrichment = 7.8055
TSS Enrichment
25
20
15
10
5
0
2.5
3.0
3.5
4.0
4.5
5.0
Log 10 (Unique Fragments)
Density
0.05
0.10
0.15
0.20
GE GW 9 b
nCells Pass Filter = 8057
Median Frags = 8786
Median TSS Enrichment = 7.482
TSS Enrichment
30
20
10
0
2.5
3.0
3.5
4.0
4.5
5.0
Log 10 (Unique Fragments)
Density
0.05
0.10
0.15
0.20

Fig. S6. Quality control results of scRNA-seq and scATAC-seq

a. Predicted doublet distribution of each scRNA-seq data. Only singlets were kept for downstream analysis.

b. Predicted doublet distribution of each scATAC-seq data. Scale bar represented doublet scores (−log10(P.adj)).

c. TSS enrichment score vs unique fragments per cell from each scATAC-seq data. Dot color represented the density in arbitrary units of points in the plot.
