# Supplementary material for: Evolutionarily conservative and non-conservative regulatory networks during primate interneuron development revealed by single-cell RNA and ATAC sequencing
Source: Cell Res. 2022 Mar 10;32(5):425–36. doi: 10.1038/s41422-022-00635-9 (PMC9061815; doi:10.1038/s41422-022-00635-9)
Supplement: Supplementary file 7 — Table S1 [file 41422_2022_635_MOESM7_ESM.pdf]

ID	Species	Time / GW	Gender	Sequencing Saturation	Estimated Number of Cells	Cells after QC	Mean Genes/Cell after QC
1	*Macaque fascicularis*	7	Male	0.581	12293	10601	1766.717
2	*Macaque fascicularis*	10	Male	0.704	13004	9504	1159.28
3	*Macaque fascicularis*	12	Male	0.724	11951	9164	1229.356
4	*Homo sapiens*	9	Female	0.7	8426	7478	2357.052
5	*Homo sapiens*	13	Male	0.834	7830	6304	2007.94
