# Supplementary material for: Evolutionarily conservative and non-conservative regulatory networks during primate interneuron development revealed by single-cell RNA and ATAC sequencing
Source: Cell Res. 2022 Mar 10;32(5):425–36. doi: 10.1038/s41422-022-00635-9 (PMC9061815; doi:10.1038/s41422-022-00635-9)
Supplement: Supplementary file 8 — Table S2 [file 41422_2022_635_MOESM8_ESM.pdf]

	p_val	avg_log2FC	pct.1	pct.2	p_val_adj
HES4	0	0.64056127	0.391	0.116	0
HES5	0	1.036634817	0.409	0.038	0
AL139246.5	0	1.077360367	0.369	0.014	0
ENO1	0	1.030093063	0.852	0.469	0
PDPN	0	0.490052391	0.35	0.02	0
HMGN2	0	2.306527043	0.996	0.884	0
RCC1	0	0.323169325	0.273	0.041	0
CLSPN	0	0.788367103	0.453	0.053	0
CDCA8	0	0.545225381	0.267	0.009	0
CDC20	0	1.139101867	0.349	0.021	0
KIF2C	0	0.484171709	0.257	0.008	0
NASP	0	1.071363389	0.901	0.609	0
CDKN2C	0	0.352041438	0.225	0.028	0
MAGOH	0	0.621677731	0.681	0.26	0
HSPB11	0	0.512538742	0.469	0.154	0
PLPP3	0	0.496772287	0.314	0.011	0
NFIA	0	0.522434691	0.703	0.271	0
USP1	0	0.825365149	0.652	0.209	0
ITGB3BP	0	0.519526404	0.418	0.071	0
AK4	0	0.256216142	0.183	0.008	0
DEPDC1	0	0.385537717	0.222	0.008	0
GNG5	0	1.477081496	0.919	0.254	0
CCN1	0	0.566036963	0.321	0.04	0
PSRC1	0	0.573744822	0.295	0.029	0
RHOC	0	0.271046015	0.209	0.017	0
SLC16A1	0	0.412310562	0.368	0.079	0
PHGDH	0	1.075843438	0.706	0.113	0
NOTCH2	0	0.250710787	0.176	0.007	0
ANP32E	0	1.502189028	0.818	0.24	0
RAB13	0	0.394184193	0.349	0.081	0
CKS1B	0	1.437494536	0.674	0.101	0
FDPS	0	0.689925998	0.762	0.418	0
NES	0	1.029794199	0.673	0.16	0
NUF2	0	1.037097348	0.422	0.019	0
ILDR2	0	0.290911392	0.22	0.024	0
PRDX6	0	0.718813542	0.668	0.223	0
ASPM	0	1.476182454	0.422	0.031	0
KIF14	0	0.326836803	0.185	0.008	0
UBE2T	0	1.19115628	0.6	0.149	0
NUCKS1	0	1.035805754	0.967	0.863	0
NEK2	0	0.55363511	0.227	0.009	0
DTL	0	0.286088856	0.198	0.007	0
CENPF	0	2.666739213	0.635	0.082	0
RPS7	0	0.467939857	0.999	0.999	0
RRM2	0	0.832664615	0.425	0.019	0
CENPA	0	0.594731661	0.253	0.007	0
ATRAID	0	0.503029301	0.513	0.156	0
ZFP36L2	0	0.675163355	0.473	0.071	0
SNRPG	0	0.611831653	0.895	0.658	0
NCAPH	0	0.364223625	0.247	0.009	0
PANTR1	0	0.960240703	0.859	0.544	0
LINC01159	0	0.433298575	0.301	0.024	0
SEPTIN10	0	0.377794135	0.338	0.074	0
BUB1	0	0.447634076	0.237	0.01	0
CKAP2L	0	0.712531448	0.347	0.01	0
DBI	0	0.72010593	0.826	0.502	0
MCM6	0	0.523015637	0.352	0.037	0

GCA	0	0.385725676	0.315	0.055	0
STK39	0	0.489967009	0.423	0.105	0
SPC25	0	0.887082416	0.439	0.022	0
HAT1	0	0.724039825	0.592	0.169	0
CDCA7	0	0.435812507	0.402	0.117	0
NFE2L2	0	0.337780745	0.265	0.037	0
GULP1	0	0.332834744	0.226	0.011	0
HSPD1	0	0.743298463	0.897	0.674	0
HSPE1	0	0.638757899	0.926	0.761	0
SGO2	0	0.926035199	0.354	0.028	0
BARD1	0	0.572844653	0.441	0.064	0
HJURP	0	0.421292478	0.247	0.007	0
HES6	0	1.023859132	0.671	0.376	0
DTYMK	0	0.873449613	0.617	0.156	0
SGO1	0	0.748282003	0.4	0.018	0
KIF15	0	0.609376434	0.353	0.015	0
RHOA	0	0.599592613	0.806	0.456	0
POC1A	0	0.27152151	0.199	0.007	0
FAM107A	0	0.516357699	0.184	0.006	0
CMSS1	0	0.440596687	0.433	0.13	0
CIP2A	0	0.475832	0.312	0.023	0
MCM2	0	0.374796368	0.268	0.025	0
CNBP	0	0.678133991	0.888	0.606	0
TFDP2	0	1.116579348	0.725	0.236	0
WWTR1	0	0.270552844	0.175	0.009	0
VEPH1	0	0.485793473	0.301	0.011	0
SMC4	0	1.72804976	0.712	0.154	0
TRIM59	0	0.854954534	0.466	0.072	0
ECT2	0	0.489624481	0.243	0.01	0
SOX2	0	0.787003404	0.952	0.625	0
RFC4	0	0.460866224	0.414	0.122	0
HES1	0	1.084550208	0.383	0.028	0
TACC3	0	0.602411481	0.328	0.013	0
NCAPG	0	0.561997078	0.327	0.018	0
RFC1	0	0.524790433	0.511	0.187	0
PAICS	0	0.788379175	0.644	0.172	0
SEPTIN11	0	0.984229905	0.882	0.528	0
PDLIM5	0	0.367885888	0.28	0.046	0
H2AFZ	0	1.582280896	0.988	0.933	0
BDH2	0	0.401743281	0.317	0.031	0
CENPE	0	1.009140812	0.33	0.022	0
HADH	0	0.372483895	0.358	0.091	0
MAD2L1	0	1.321577967	0.622	0.053	0
ANXA5	0	0.915108403	0.645	0.074	0
CCNA2	0	0.823108594	0.365	0.019	0
MND1	0	0.541758209	0.372	0.019	0
SFRP2	0	0.79930256	0.485	0.034	0
TMA16	0	0.561163743	0.436	0.076	0
HMGB2	0	2.814444456	0.853	0.286	0
CENPU	0	0.766152942	0.487	0.048	0
PDLIM3	0	0.551334423	0.397	0.038	0
SNHG18	0	0.299151091	0.198	0.015	0
MYO10	0	0.489723161	0.387	0.066	0
RAI14	0	0.428343156	0.311	0.045	0
SLC1A3	0	0.5643595	0.259	0.023	0
DEPDC1B	0	0.479222725	0.319	0.026	0
ADAMTS6	0	0.291561026	0.218	0.02	0
CENPK	0	0.708574459	0.447	0.025	0

CCNB1	0	1.351892335	0.347	0.032	0
CENPH	0	0.605355981	0.494	0.1	0
IQGAP2	0	0.353314178	0.233	0.016	0
DHFR	0	0.849936066	0.481	0.057	0
ADGRV1	0	1.053394079	0.59	0.123	0
LIX1	0	0.860482836	0.384	0.016	0
LMNB1	0	0.644854869	0.572	0.217	0
H2AFY	0	0.618096079	0.899	0.697	0
KIF20A	0	0.339977495	0.192	0.005	0
CDC25C	0	0.301137958	0.192	0.005	0
CXXC5	0	0.376412375	0.366	0.085	0
SPARC	0	0.881398826	0.458	0.029	0
G3BP1	0	0.517881466	0.625	0.283	0
PTTG1	0	2.063984745	0.628	0.119	0
NUDCD2	0	0.61544663	0.568	0.231	0
HMMR	0	0.363439874	0.194	0.007	0
NPM1	0	0.678959783	0.993	0.974	0
MXD3	0	0.623176457	0.325	0.014	0
DEK	0	1.420366054	0.973	0.69	0
E2F3	0	0.332185106	0.25	0.033	0
GMNN	0	0.913026903	0.601	0.089	0
HIST1H1A	0	0.903315611	0.418	0.095	0
HIST1H3B	0	1.69150606	0.398	0.035	0
HIST1H3C	0	0.290405803	0.167	0.007	0
HIST1H1C	0	1.668616064	0.657	0.252	0
HIST1H4C	0	3.343301911	0.874	0.782	0
HIST1H1E	0	1.035327192	0.538	0.223	0
HIST1H3D	0	1.472104686	0.406	0.113	0
HIST1H1D	0	1.497827327	0.544	0.127	0
HIST1H3F	0	0.474539317	0.194	0.007	0
HIST1H3G	0	0.552182514	0.25	0.007	0
HIST1H2AG	0	1.220513206	0.385	0.066	0
HIST1H2AH	0	0.42679967	0.218	0.012	0
HIST1H2AI	0	0.345989111	0.181	0.012	0
HIST1H2AJ	0	0.665397123	0.294	0.022	0
HIST1H2AL	0	0.71869307	0.276	0.025	0
HIST1H1B	0	1.654701162	0.42	0.05	0
CLIC1	0	0.867304512	0.733	0.202	0
KIFC1	0	0.641755446	0.359	0.011	0
UQCC2	0	0.629762667	0.635	0.248	0
SRSF3	0	0.636684165	0.975	0.871	0
GLO1	0	0.793832031	0.706	0.233	0
DNPH1	0	0.518115401	0.403	0.072	0
CENPQ	0	0.332273025	0.267	0.03	0
MCM3	0	0.578287173	0.379	0.039	0
HMGN3	0	1.115315431	0.83	0.352	0
TTK	0	0.537051079	0.273	0.01	0
MMS22L	0	0.539479616	0.413	0.09	0
NR2E1	0	0.318692996	0.244	0.027	0
FABP7	0	2.163384355	0.453	0.141	0
CENPW	0	0.572718436	0.418	0.067	0
AHI1	0	0.539811136	0.43	0.08	0
FBXO5	0	0.87631637	0.483	0.047	0
RPA3	0	0.78854617	0.624	0.175	0
CDCA7L	0	0.384175342	0.321	0.057	0
HNRNPA2B1	0	0.693962339	0.998	0.993	0
CREB5	0	0.741706978	0.443	0.038	0
GGCT	0	0.536982888	0.434	0.118	0

ANLN	0	0.278195839	0.192	0.01	0
GLI3	0	0.553656775	0.321	0.025	0
PPIA	0	0.798728903	0.999	0.982	0
H2AFV	0	1.190135589	0.904	0.501	0
HSPB1	0	0.874422594	0.767	0.353	0
MAGI2-AS3	0	0.385494145	0.32	0.06	0
DBF4	0	0.729590326	0.459	0.082	0
SRI	0	0.900732191	0.835	0.402	0
CDK6	0	0.905525361	0.521	0.065	0
PON2	0	0.67138773	0.404	0.039	0
SEM1	0	0.74782421	0.9	0.612	0
MCM7	0	0.971311374	0.662	0.188	0
TRIP6	0	0.275602984	0.2	0.012	0
LRRC17	0	0.351869421	0.239	0.021	0
SMIM30	0	0.355535339	0.281	0.043	0
PTPRZ1	0	1.429442608	0.699	0.23	0
AASS	0	0.468455787	0.369	0.057	0
CALD1	0	0.582099394	0.53	0.154	0
RARRES2	0	0.303207959	0.191	0.008	0
RHEB	0	0.784589883	0.776	0.373	0
XRCC2	0	0.386041655	0.247	0.013	0
SLC25A37	0	0.459947424	0.369	0.043	0
CDCA2	0	0.335800928	0.205	0.004	0
CLU	0	0.828376722	0.44	0.059	0
ESCO2	0	0.518130859	0.313	0.012	0
PBK	0	1.2856055	0.511	0.023	0
FGFR1	0	0.414847571	0.334	0.063	0
SFRP1	0	1.387439082	0.667	0.208	0
GINS4	0	0.252441264	0.177	0.008	0
MCM4	0	0.538643899	0.368	0.06	0
SNHG6	0	0.670818374	0.957	0.82	0
CCNE2	0	0.362445318	0.261	0.043	0
LAPTM4B	0	0.503609251	0.48	0.14	0
DSCC1	0	0.251230967	0.185	0.012	0
ATAD2	0	0.791359765	0.469	0.041	0
EEF1D	0	0.951811685	0.934	0.708	0
RPL8	0	0.551816006	0.999	0.999	0
CNTLN	0	0.462190004	0.356	0.058	0
RPS6	0	0.549455693	0.999	0.999	0
MTAP	0	0.38778786	0.341	0.072	0
TPM2	0	0.489256177	0.343	0.045	0
MELK	0	0.369494732	0.253	0.009	0
PSAT1	0	0.445908948	0.347	0.061	0
CKS2	0	1.552639241	0.639	0.12	0
ANP32B	0	1.063806469	0.843	0.381	0
SMC2	0	1.082058524	0.634	0.086	0
CTNNAL1	0	0.608562947	0.429	0.05	0
PHF19	0	0.449772837	0.323	0.02	0
CRB2	0	0.254071755	0.165	0.005	0
LHX2	0	0.413614199	0.288	0.038	0
WDR34	0	0.481553736	0.421	0.09	0
AIF1L	0	0.529221408	0.405	0.045	0
NOTCH1	0	0.574956307	0.418	0.054	0
TUBB4B	0	1.537248196	0.708	0.421	0
MCM10	0	0.376036426	0.25	0.008	0
VIM	0	2.888627422	0.864	0.452	0
ZWINT	0	0.783686947	0.475	0.019	0
CDK1	0	1.401511479	0.502	0.031	0

RTKN2	0	0.629732643	0.404	0.053	0
PCBD1	0	0.448856576	0.412	0.095	0
DNAJC9	0	0.56482314	0.509	0.157	0
KIF20B	0	0.752885786	0.424	0.104	0
FGFBP3	0	0.56691999	0.338	0.062	0
KIF11	0	0.821040807	0.402	0.02	0
CEP55	0	0.363999601	0.208	0.006	0
HELLS	0	0.941993143	0.584	0.123	0
PDLIM1	0	0.276506167	0.212	0.023	0
TCF7L2	0	0.41381763	0.319	0.047	0
BUB3	0	0.709023282	0.619	0.288	0
MKI67	0	1.707875514	0.531	0.032	0
RRM1	0	0.737270714	0.572	0.164	0
WEE1	0	0.416448873	0.338	0.058	0
LDHA	0	1.186290518	0.817	0.332	0
CCDC34	0	0.786549857	0.529	0.114	0
KIF18A	0	0.408764245	0.221	0.007	0
RCN1	0	0.71072978	0.528	0.089	0
MDK	0	1.365466563	0.846	0.271	0
FAM111B	0	0.308604504	0.21	0.009	0
FAM111A	0	0.295606646	0.196	0.01	0
FEN1	0	0.41470685	0.309	0.044	0
CDCA5	0	0.329961432	0.232	0.008	0
RNASEH2C	0	0.523830959	0.476	0.127	0
BANF1	0	0.583390978	0.878	0.652	0
GSTP1	0	1.288037266	0.974	0.679	0
CCND1	0	1.114294848	0.414	0.056	0
POLD3	0	0.37754354	0.314	0.066	0
YAP1	0	0.267811315	0.192	0.007	0
H2AFX	0	1.312032189	0.623	0.099	0
SPA17	0	0.347824968	0.246	0.032	0
CHEK1	0	0.675339604	0.449	0.029	0
FOXM1	0	0.435730555	0.294	0.01	0
CCND2	0	1.102108266	0.822	0.448	0
RAD51AP1	0	0.680600537	0.425	0.02	0
NCAPD2	0	0.420145682	0.293	0.042	0
GAPDH	0	0.942176482	0.999	0.996	0
CDCA3	0	0.844281068	0.336	0.019	0
TPI1	0	1.194298342	0.917	0.509	0
YBX3	0	0.31242873	0.219	0.011	0
APOLD1	0	0.467640847	0.3	0.04	0
DERA	0	0.380508952	0.33	0.06	0
MGST1	0	0.49947632	0.255	0.01	0
LDHB	0	0.707386391	0.99	0.927	0
SCAF11	0	0.528309138	0.537	0.191	0
TMEM106C	0	0.600182956	0.535	0.163	0
TUBA1B	0	2.273442867	0.982	0.931	0
TROAP	0	0.435906468	0.23	0.011	0
RACGAP1	0	0.564943794	0.332	0.034	0
CDK2	0	0.260685245	0.194	0.016	0
RPL41	0	0.615914053	0.999	1	0
CDK4	0	0.768513498	0.679	0.248	0
HMGA2	0	0.5975185	0.36	0.023	0
NAP1L1	0	0.817110704	0.979	0.831	0
CSRP2	0	0.470279894	0.709	0.339	0
NEDD1	0	0.300728233	0.252	0.034	0
TMPO	0	0.864674504	0.789	0.467	0
PARPBP	0	0.313240169	0.207	0.01	0

HSP90B1	0	0.866950768	0.725	0.39	0
RPLP0	0	0.561021577	0.999	0.997	0
CDK2AP1	0	0.623649526	0.626	0.252	0
RAN	0	1.071330352	0.987	0.883	0
PXMP2	0	0.483434126	0.438	0.132	0
SKA3	0	0.292080419	0.207	0.01	0
HMGB1	0	1.43175492	1	0.998	0
BRCA2	0	0.385447406	0.255	0.012	0
RFC3	0	0.413788492	0.344	0.068	0
EXOSC8	0	0.591600281	0.646	0.269	0
CKAP2	0	1.084619284	0.484	0.066	0
DIAPH3	0	0.307675591	0.219	0.009	0
SOX1-OT	0	0.438148659	0.396	0.111	0
CCNB1IP1	0	0.47694648	0.508	0.184	0
MIS18BP1	0	0.994061441	0.527	0.062	0
LRR1	0	0.373412118	0.292	0.027	0
TMX1	0	0.421084573	0.436	0.127	0
CDKN3	0	0.925018878	0.38	0.019	0
DLGAP5	0	0.913845336	0.337	0.013	0
ZFP36L1	0	1.284246351	0.648	0.047	0
ERH	0	0.591890148	0.978	0.869	0
CKB	0	0.873416339	0.997	0.994	0
SIVA1	0	0.854313177	0.719	0.237	0
CDCA4	0	0.459845383	0.363	0.063	0
TJP1	0	0.461942623	0.391	0.07	0
ARHGAP11A	0	0.517568351	0.299	0.025	0
BUB1B	0	0.448936264	0.255	0.01	0
KNSTRN	0	0.670562354	0.324	0.073	0
KNL1	0	0.689014978	0.352	0.02	0
OIP5	0	0.317782741	0.223	0.005	0
NUSAP1	0	2.051812173	0.624	0.054	0
SERF2	0	0.585651407	0.984	0.899	0
WDR76	0	0.379275125	0.305	0.051	0
B2M	0	0.393585612	0.398	0.074	0
DUT	0	1.418893726	0.767	0.308	0
CEP152	0	0.290547166	0.209	0.016	0
MNS1	0	0.378321779	0.236	0.016	0
CCNB2	0	1.306926734	0.474	0.046	0
ANXA2	0	0.383133131	0.233	0.028	0
RPS27L	0	0.67217942	0.742	0.405	0
PCLAF	0	1.540758854	0.593	0.051	0
KIF23	0	0.49706443	0.256	0.007	0
RPLP1	0	0.740939006	1	1	0
UACA	0	0.257607051	0.175	0.01	0
PKM	0	0.894528708	0.834	0.433	0
MFGE8	0	0.341052975	0.257	0.026	0
FANCI	0	0.344436577	0.25	0.019	0
PRC1	0	1.122609401	0.478	0.057	0
NME4	0	0.683782245	0.626	0.23	0
METRN	0	0.564009201	0.35	0.023	0
RPS2	0	0.80983132	0.999	1	0
ECI1	0	0.463785986	0.404	0.101	0
PKMYT1	0	0.262977443	0.183	0.009	0
EMP2	0	0.308936597	0.215	0.008	0
RMI2	0	0.262680601	0.19	0.013	0
LITAF	0	0.370015491	0.247	0.033	0
PLK1	0	0.653090198	0.234	0.012	0
KIF22	0	0.769431527	0.473	0.089	0

ORC6	0	0.763814522	0.511	0.059	0
TOX3	0	0.547829919	0.528	0.18	0
MT3	0	0.492576782	0.282	0.041	0
MT2A	0	0.677107014	0.389	0.03	0
CKLF	0	0.473118422	0.45	0.127	0
CENPN	0	0.451821884	0.32	0.021	0
GCSH	0	0.667760575	0.628	0.273	0
GINS2	0	0.983013604	0.559	0.089	0
CDT1	0	0.382561404	0.257	0.018	0
PFN1	0	0.664240912	0.861	0.573	0
PIMREG	0	0.859800083	0.393	0.018	0
NAA38	0	0.522740599	0.66	0.313	0
AURKB	0	0.904403589	0.411	0.031	0
SPAG5	0	0.289649302	0.2	0.01	0
TP53I13	0	0.366382506	0.291	0.024	0
ATAD5	0	0.435464611	0.363	0.094	0
TMEM98	0	0.558806738	0.6	0.221	0
TOP2A	0	2.69438887	0.611	0.072	0
BRCA1	0	0.493665886	0.324	0.016	0
NME2	0	0.777901017	0.85	0.578	0
PRR11	0	0.267075008	0.18	0.012	0
SOX9	0	0.770034621	0.6	0.228	0
SRSF2	0	0.707780106	0.86	0.566	0
TK1	0	0.400412571	0.261	0.009	0
BIRC5	0	1.43826685	0.558	0.038	0
DCXR	0	0.899212742	0.602	0.144	0
TYMSOS	0	0.280268591	0.194	0.012	0
TYMS	0	1.352073566	0.58	0.048	0
NDC80	0	0.711559104	0.37	0.013	0
TGIF1	0	0.319903446	0.242	0.034	0
IMPA2	0	0.307243049	0.234	0.016	0
TUBB6	0	0.386638991	0.245	0.01	0
SNRPD1	0	0.622233092	0.906	0.714	0
RBBP8	0	0.326788364	0.252	0.035	0
ACAA2	0	0.540572043	0.483	0.108	0
SALL3	0	0.363176377	0.243	0.016	0
BSG	0	0.608979614	0.733	0.388	0
PTBP1	0	0.456466721	0.423	0.097	0
RPS15	0	0.509722098	1	1	0
CHAF1A	0	0.34416792	0.272	0.037	0
UHRF1	0	0.476323013	0.345	0.032	0
SPC24	0	0.334369759	0.239	0.019	0
RNASEH2A	0	0.383745568	0.305	0.041	0
ASF1B	0	0.265298496	0.186	0.006	0
DDX39A	0	0.571956038	0.535	0.218	0
NOTCH3	0	0.265347698	0.191	0.009	0
LSM4	0	0.842040947	0.8	0.397	0
C19orf48	0	0.589832362	0.476	0.096	0
TTYH1	0	1.677044392	0.661	0.162	0
SNRPB	0	1.158720672	0.907	0.476	0
CDC25B	0	0.457479043	0.291	0.023	0
PCNA	0	1.064740287	0.606	0.136	0
MGME1	0	0.478947172	0.398	0.074	0
TPX2	0	1.313679953	0.451	0.026	0
AHCY	0	0.391664962	0.369	0.097	0
DSN1	0	0.349451174	0.252	0.016	0
MYBL2	0	0.410038502	0.255	0.011	0
UBE2C	0	2.356080945	0.532	0.073	0

AURKA	0	0.491512786	0.23	0.018	0
BTG3	0	0.951703326	0.737	0.22	0
JAM2	0	0.431617792	0.348	0.039	0
OLIG2	0	0.377025369	0.245	0.022	0
OLIG1	0	0.352496654	0.212	0.014	0
C21orf58	0	0.727521204	0.431	0.034	0
CDC45	0	0.361185559	0.242	0.011	0
RANBP1	0	1.067772621	0.937	0.681	0
MCM5	0	0.530785414	0.373	0.056	0
CENPM	0	0.680298737	0.422	0.02	0
FBLN1	0	0.52932029	0.544	0.197	0
GTSE1	0	0.971985177	0.407	0.016	0
CD99	0	0.71819421	0.531	0.091	0
RBBP7	0	0.526220439	0.529	0.168	0
PRDX4	0	0.68023546	0.558	0.132	0
KIF4A	0	0.478599789	0.269	0.011	0
HMGN5	0	0.60606674	0.431	0.064	0
SH3BGRL	0	0.597166672	0.746	0.379	0
PMAIP1	5.31E-308	0.269381119	0.19	0.016	1.12E-303
MIR99AHG	2.26E-307	0.690319853	0.664	0.329	4.77E-303
LINC01896	3.71E-307	0.297282572	0.201	0.02	7.84E-303
PA2G4	5.81E-307	0.634527197	0.783	0.491	1.23E-302
ITGB8	1.87E-306	0.493590514	0.382	0.108	3.96E-302
SALL1	3.68E-306	0.295314737	0.233	0.031	7.77E-302
PTMA	5.98E-306	0.459602331	1	1	1.26E-301
NUP37	2.35E-305	0.301364994	0.263	0.044	4.96E-301
RPL22L1	2.85E-304	0.515879114	0.498	0.19	6.02E-300
HIST1H2BH	4.40E-304	0.460862159	0.286	0.057	9.31E-300
TOMM5	1.81E-303	0.594376374	0.786	0.488	3.83E-299
MZT1	3.40E-303	0.62363017	0.448	0.157	7.18E-299
TMEM97	7.99E-301	0.510732333	0.554	0.228	1.69E-296
HNRNPA3	2.53E-299	0.54311029	0.971	0.901	5.35E-295
PLIN3	4.49E-299	0.277959665	0.205	0.022	9.49E-295
TAGLN2	4.14E-298	0.314886314	0.206	0.023	8.76E-294
STAG2	2.10E-297	0.454526438	0.506	0.191	4.45E-293
E2F1	1.98E-296	0.273742946	0.183	0.015	4.18E-292
TXNDC12	2.91E-296	0.39588046	0.4	0.12	6.16E-292
PDIA6	6.35E-296	0.572892074	0.687	0.364	1.34E-291
GPM6B	1.00E-295	0.788489322	0.708	0.419	2.12E-291
FZD2	1.19E-295	0.387771	0.395	0.116	2.52E-291
TMBIM6	1.64E-295	0.582062977	0.828	0.561	3.47E-291
ATP1A2	6.93E-294	0.317386418	0.152	0.006	1.47E-289
HIST1H2BB	7.65E-294	0.521165783	0.196	0.02	1.62E-289
ARL6IP6	1.28E-293	0.53290071	0.637	0.314	2.70E-289
HDGF	9.45E-293	0.561266498	0.559	0.247	2.00E-288
LSM5	3.71E-289	0.678005038	0.761	0.471	7.85E-285
FZD8	9.61E-289	0.328364559	0.196	0.021	2.03E-284
COMMD4	1.13E-288	0.42770868	0.479	0.176	2.38E-284
ANP32A	1.13E-288	0.543820329	0.883	0.638	2.38E-284
RORB	7.33E-288	0.426020387	0.375	0.107	1.55E-283
JPT2	8.48E-288	0.354046517	0.361	0.099	1.79E-283
CRYGD	2.40E-287	0.306890459	0.226	0.032	5.07E-283
EEF1B2	1.06E-286	0.591248799	0.918	0.744	2.24E-282
S100A6	3.40E-285	0.285903844	0.252	0.043	7.19E-281
RPL12	9.73E-285	0.55340602	0.995	0.991	2.06E-280
RNASEH2B	2.28E-284	0.60705899	0.606	0.296	4.82E-280
MIS18A	2.30E-284	0.396299534	0.385	0.115	4.86E-280
PSMG1	5.87E-284	0.299329598	0.251	0.044	1.24E-279

ASCL1	4.17E-283	1.010509851	0.648	0.355	8.82E-279
PEX2	7.88E-283	0.330404928	0.305	0.07	1.67E-278
PEA15	4.65E-282	0.640965421	0.54	0.244	9.83E-278
TGIF2	7.61E-282	0.261700955	0.214	0.028	1.61E-277
TEAD2	8.08E-282	0.314157623	0.286	0.06	1.71E-277
HNRNPF	1.06E-281	0.538621803	0.769	0.469	2.23E-277
RFC5	1.24E-281	0.297047138	0.245	0.042	2.62E-277
MFAP2	2.61E-281	0.331087123	0.263	0.05	5.51E-277
FABP5	1.23E-280	0.96227395	0.888	0.734	2.60E-276
ZEB1	2.74E-280	0.475434433	0.48	0.18	5.80E-276
CENPL	3.29E-280	0.274420825	0.2	0.024	6.96E-276
FAM83D	1.44E-279	0.261581511	0.158	0.01	3.05E-275
PLIN2	3.09E-279	0.342761305	0.332	0.085	6.54E-275
MRPL11	1.72E-278	0.457941516	0.533	0.224	3.64E-274
SRSF7	3.76E-277	0.552979006	0.924	0.778	7.96E-273
ANAPC11	9.67E-277	0.570916368	0.865	0.643	2.04E-272
LMNB2	5.03E-276	0.36019692	0.327	0.084	1.06E-271
SPTSSA	1.56E-275	0.333576486	0.33	0.085	3.30E-271
SEMA5A	3.55E-275	0.344762061	0.276	0.057	7.51E-271
DPM3	1.46E-274	0.406369543	0.44	0.154	3.08E-270
SNRPD3	1.48E-274	0.496682439	0.783	0.482	3.14E-270
MRPL51	1.27E-273	0.559750703	0.839	0.59	2.68E-269
GSX2	1.27E-273	0.360015411	0.25	0.045	2.69E-269
SAE1	2.35E-272	0.435819136	0.473	0.181	4.97E-268
EZR	6.30E-272	0.413443703	0.461	0.165	1.33E-267
MZT2B	9.21E-272	0.506462268	0.952	0.841	1.95E-267
FGFR3	3.15E-271	0.285913548	0.156	0.01	6.65E-267
HIST1H2AB	4.23E-271	0.327204405	0.167	0.014	8.95E-267
NENF	4.83E-270	0.34513462	0.298	0.07	1.02E-265
INTU	1.07E-269	0.515075091	0.408	0.14	2.27E-265
HNRNPD	1.74E-269	0.546005402	0.943	0.822	3.68E-265
GSTO1	3.97E-269	0.295333504	0.27	0.056	8.39E-265
PTGES3	5.96E-269	0.504163128	0.943	0.828	1.26E-264
RPA2	8.85E-269	0.430588711	0.439	0.16	1.87E-264
CD63	3.50E-268	0.580126538	0.731	0.445	7.41E-264
LIMS1	8.39E-268	0.351691453	0.354	0.1	1.77E-263
EFCAB2	1.40E-267	0.2984735	0.265	0.054	2.95E-263
TMEM14C	3.36E-267	0.471566234	0.604	0.287	7.11E-263
GPSM2	2.18E-266	0.504371908	0.418	0.149	4.60E-262
LIG1	1.62E-265	0.317071523	0.266	0.055	3.43E-261
BBX	6.98E-265	0.404794679	0.406	0.134	1.48E-260
OSTC	4.80E-264	0.470310232	0.612	0.299	1.02E-259
CMTM6	1.19E-263	0.339468032	0.341	0.095	2.52E-259
TSPAN6	3.72E-263	0.392874364	0.412	0.14	7.87E-259
CD82	4.56E-263	0.253664133	0.139	0.006	9.64E-259
TCIM	5.31E-262	0.267573621	0.152	0.01	1.12E-257
RPL23A	2.73E-261	0.421456225	0.998	0.997	5.77E-257
SSB	3.38E-261	0.548740972	0.833	0.584	7.14E-257
PSME1	2.45E-260	0.412514564	0.481	0.188	5.18E-256
DCTPP1	1.05E-259	0.463232631	0.452	0.175	2.22E-255
NDE1	5.17E-259	0.28588873	0.194	0.025	1.09E-254
RBMX	5.91E-259	0.539404359	0.937	0.814	1.25E-254
AC011447.3	1.04E-258	0.296656867	0.25	0.049	2.19E-254
RAD21	2.23E-258	0.767687777	0.726	0.473	4.71E-254
MID1	1.03E-257	0.309293334	0.268	0.057	2.17E-253
ENY2	2.76E-257	0.519465412	0.814	0.545	5.83E-253
LMO1	6.88E-257	0.343601162	0.218	0.035	1.46E-252
CNN3	2.65E-256	0.561088451	0.743	0.44	5.61E-252

MPP5	2.70E-256	0.265984633	0.22	0.036	5.71E-252
HIST2H2AC	7.54E-256	0.749580534	0.365	0.12	1.59E-251
HIST1H4H	2.81E-255	0.372341862	0.183	0.022	5.94E-251
RPL35	3.82E-255	0.475328467	0.997	0.99	8.08E-251
CMC2	7.15E-255	0.486357851	0.515	0.229	1.51E-250
QKI	7.52E-255	0.603580157	0.758	0.472	1.59E-250
LINC01551	2.16E-253	0.695885198	0.762	0.478	4.58E-249
JADE1	2.23E-253	0.37855405	0.344	0.101	4.72E-249
TRMT112	2.39E-253	0.49397928	0.797	0.504	5.06E-249
SPART	2.78E-253	0.331298864	0.3	0.076	5.89E-249
WDHD1	6.43E-253	0.265090955	0.213	0.034	1.36E-248
LSM2	2.96E-250	0.463911771	0.7	0.396	6.27E-246
SNRPA	7.66E-250	0.42686681	0.554	0.254	1.62E-245
POLD2	5.61E-249	0.438005936	0.551	0.253	1.19E-244
UCP2	2.41E-248	0.323675675	0.309	0.082	5.10E-244
RFC2	4.48E-248	0.389496861	0.37	0.121	9.48E-244
RPA1	4.72E-248	0.283525897	0.263	0.057	9.97E-244
KPNA2	8.99E-248	1.363123849	0.555	0.294	1.90E-243
GGH	3.24E-247	0.363921412	0.401	0.138	6.84E-243
CEP135	6.99E-247	0.297126461	0.26	0.056	1.48E-242
POLR3K	9.36E-247	0.322005397	0.318	0.088	1.98E-242
SMS	9.71E-247	0.636139016	0.692	0.399	2.05E-242
PNRC2	1.37E-246	0.477147838	0.578	0.282	2.89E-242
SNRPA1	2.71E-246	0.42136687	0.592	0.284	5.73E-242
CALU	5.87E-246	0.405461374	0.452	0.176	1.24E-241
NME1	1.13E-245	0.604944622	0.748	0.491	2.39E-241
DARS	1.54E-245	0.426080172	0.46	0.182	3.25E-241
IKBIP	1.36E-244	0.262462559	0.219	0.038	2.88E-240
LBR	1.41E-244	0.576680257	0.607	0.321	2.99E-240
YEATS4	2.18E-244	0.390895558	0.443	0.167	4.60E-240
PPIF	4.82E-244	0.286820676	0.267	0.06	1.02E-239
ZNF511	4.32E-242	0.298393001	0.293	0.075	9.13E-238
SNX5	4.65E-242	0.332715401	0.338	0.1	9.84E-238
TMEM170A	1.41E-241	0.32207172	0.313	0.086	2.97E-237
ALYREF	4.95E-241	0.423075139	0.371	0.125	1.05E-236
DNAJC1	9.26E-241	0.383536173	0.311	0.086	1.96E-236
EZH2	1.11E-240	0.569152224	0.631	0.349	2.34E-236
NSMCE4A	2.74E-240	0.379507641	0.427	0.161	5.79E-236
OTX2	2.87E-240	0.270596879	0.18	0.023	6.06E-236
CMBL	2.97E-240	0.306789663	0.272	0.064	6.27E-236
TEX30	3.29E-240	0.270528668	0.26	0.058	6.95E-236
CDO1	1.18E-239	0.618826404	0.489	0.22	2.49E-235
CLNS1A	2.04E-237	0.462920677	0.686	0.394	4.32E-233
TUBA1C	2.26E-237	0.938851616	0.38	0.138	4.77E-233
NT5C	6.05E-237	0.392597363	0.469	0.193	1.28E-232
SCRG1	3.37E-236	0.329781631	0.225	0.043	7.13E-232
NAA50	1.19E-235	0.35021237	0.37	0.123	2.52E-231
PARP1	7.81E-235	0.513586775	0.755	0.48	1.65E-230
MAGOHB	1.59E-234	0.364999068	0.412	0.151	3.37E-230
ATP1B3	1.80E-234	0.549979002	0.618	0.327	3.81E-230
SINHCAF	2.35E-233	0.451147131	0.585	0.287	4.97E-229
RAB31	2.74E-233	0.340891723	0.248	0.055	5.79E-229
CNTRL	3.06E-233	0.412164502	0.358	0.119	6.48E-229
NMU	7.17E-233	0.28417496	0.159	0.017	1.52E-228
SCD	2.70E-231	0.475154647	0.466	0.193	5.70E-227
CHRAC1	3.70E-231	0.284564549	0.271	0.066	7.83E-227
UQCC3	4.94E-231	0.319520538	0.355	0.114	1.04E-226
RIDA	1.30E-230	0.278908592	0.26	0.061	2.76E-226

TIPIN	3.57E-230	0.273957418	0.248	0.055	7.56E-226
SKP2	6.97E-230	0.25832464	0.235	0.049	1.47E-225
DNMT1	1.31E-229	0.461892739	0.487	0.215	2.78E-225
PSIP1	7.40E-229	0.561349784	0.786	0.542	1.57E-224
AAMDC	9.17E-229	0.262871276	0.241	0.052	1.94E-224
NUDT5	3.10E-228	0.383274249	0.52	0.232	6.55E-224
HIST1H4D	3.13E-228	0.504575351	0.228	0.048	6.62E-224
ADK	3.88E-227	0.299226711	0.283	0.074	8.22E-223
RPN2	2.47E-226	0.481202095	0.622	0.335	5.22E-222
HNRNPC	8.86E-226	0.462049877	0.952	0.843	1.87E-221
SMC1A	1.88E-225	0.482124216	0.506	0.231	3.97E-221
NCAPD3	3.63E-225	0.259841617	0.225	0.045	7.69E-221
HSDL2	7.37E-224	0.273715369	0.266	0.066	1.56E-219
CENPC	7.40E-224	0.381870241	0.363	0.125	1.57E-219
SHMT2	1.67E-223	0.279338654	0.257	0.061	3.53E-219
HNRNPAB	2.85E-223	0.516837797	0.724	0.452	6.03E-219
GPX7	8.00E-223	0.30569289	0.313	0.092	1.69E-218
AK2	3.54E-222	0.332606772	0.382	0.135	7.48E-218
STOML2	4.94E-222	0.402846798	0.57	0.282	1.05E-217
NOP56	7.55E-222	0.440355787	0.599	0.312	1.60E-217
LIMCH1	2.75E-220	0.319160977	0.237	0.052	5.81E-216
CHCHD3	2.97E-220	0.384809317	0.527	0.246	6.29E-216
CCDC167	1.87E-219	0.448466998	0.66	0.37	3.95E-215
DESI2	4.81E-219	0.318096487	0.348	0.115	1.02E-214
SRSF10	1.23E-218	0.471499809	0.866	0.659	2.61E-214
KPNB1	2.76E-218	0.457601479	0.656	0.366	5.84E-214
HIRIP3	6.08E-218	0.394875339	0.409	0.16	1.29E-213
COPRS	1.06E-217	0.30695212	0.326	0.102	2.24E-213
LARP7	2.76E-217	0.438016153	0.541	0.255	5.83E-213
CCNA1	3.68E-217	0.319937842	0.144	0.014	7.78E-213
MRPL17	4.20E-217	0.307523799	0.339	0.11	8.87E-213
MTHFD1	5.75E-217	0.255415753	0.233	0.051	1.22E-212
GKAP1	6.95E-217	0.34158825	0.363	0.125	1.47E-212
FAT1	2.28E-216	0.310749742	0.309	0.091	4.82E-212
GMPS	6.68E-215	0.362806533	0.42	0.166	1.41E-210
GPC4	9.51E-215	0.262420579	0.226	0.048	2.01E-210
PSMB2	3.14E-214	0.455220095	0.773	0.512	6.63E-210
RDX	4.70E-214	0.509050626	0.856	0.629	9.94E-210
FANCL	3.12E-213	0.306480982	0.329	0.105	6.60E-209
CCT5	2.77E-212	0.494161829	0.822	0.584	5.86E-208
ALDH9A1	1.34E-211	0.306699393	0.322	0.102	2.84E-207
OSBPL1A	2.38E-211	0.271603469	0.241	0.057	5.03E-207
LRRCC1	4.02E-211	0.267957539	0.239	0.055	8.50E-207
FAM161A	7.29E-211	0.355553362	0.35	0.12	1.54E-206
MAT2B	2.67E-210	0.31973846	0.358	0.124	5.64E-206
ELP5	6.79E-210	0.313039237	0.365	0.13	1.44E-205
SRSF1	1.30E-209	0.417625097	0.635	0.35	2.75E-205
TECR	7.02E-209	0.365289678	0.502	0.23	1.48E-204
MRPL57	1.06E-208	0.431596312	0.684	0.399	2.25E-204
PDIA3	1.25E-208	0.435629737	0.589	0.31	2.64E-204
TMEM107	1.90E-208	0.278261701	0.257	0.066	4.02E-204
PPAT	4.97E-208	0.268534082	0.245	0.06	1.05E-203
COL11A1	8.28E-207	0.287151644	0.21	0.043	1.75E-202
SERBP1	8.62E-207	0.521271853	0.914	0.786	1.82E-202
IFT57	2.71E-206	0.383827013	0.402	0.159	5.74E-202
PALLD	6.84E-206	0.288791417	0.269	0.073	1.45E-201
ASRGL1	2.62E-205	0.413335272	0.543	0.268	5.54E-201
NDUFA6	6.26E-205	0.473784365	0.787	0.546	1.32E-200

HLA-A	9.16E-205	0.308240214	0.352	0.122	1.94E-200
MPST	2.05E-204	0.358411104	0.465	0.204	4.34E-200
REST	2.29E-204	0.258072824	0.244	0.059	4.84E-200
SEPTIN2	2.95E-204	0.442461687	0.699	0.425	6.23E-200
AKR7A2	1.20E-203	0.34221473	0.402	0.159	2.55E-199
CD151	1.22E-203	0.31257745	0.347	0.12	2.58E-199
ZCCHC9	1.95E-203	0.264998864	0.251	0.064	4.12E-199
PRKDC	1.92E-202	0.563226557	0.736	0.498	4.06E-198
LSM3	2.16E-202	0.459585216	0.778	0.532	4.56E-198
SNRPC	1.36E-200	0.423150948	0.758	0.495	2.87E-196
LINC01224	1.38E-200	0.257990603	0.231	0.054	2.93E-196
HAUS1	1.60E-200	0.389351964	0.527	0.258	3.38E-196
DOK5	2.24E-200	0.368798208	0.23	0.055	4.74E-196
GPC3	4.32E-200	0.290205593	0.111	0.006	9.13E-196
GNG10	2.51E-199	0.358153908	0.476	0.214	5.32E-195
NCL	2.52E-199	0.525578401	0.913	0.78	5.33E-195
CNIH4	5.45E-199	0.400088943	0.461	0.209	1.15E-194
HMG20B	5.69E-199	0.291942303	0.327	0.109	1.20E-194
CDK5RAP2	7.20E-199	0.352914635	0.353	0.129	1.52E-194
HMGB3	1.23E-197	0.605747193	0.853	0.697	2.59E-193
ZNRD1	5.76E-197	0.317778651	0.4	0.159	1.22E-192
PGAM1	9.77E-197	0.467567642	0.713	0.464	2.07E-192
MPP6	4.18E-196	0.287353377	0.315	0.102	8.85E-192
NAE1	1.09E-195	0.384262836	0.523	0.258	2.30E-191
NELFE	3.08E-195	0.386725205	0.579	0.307	6.51E-191
IPO5	5.22E-195	0.346791614	0.42	0.176	1.10E-190
SEC11A	1.05E-194	0.437637202	0.658	0.392	2.21E-190
SOX1	1.69E-194	0.359726201	0.5	0.226	3.58E-190
ADD3	4.61E-194	0.254549001	0.223	0.052	9.75E-190
XPO1	4.78E-194	0.427461832	0.611	0.343	1.01E-189
LSM6	1.01E-193	0.377591226	0.573	0.3	2.13E-189
OLMALINC	1.17E-193	0.258298111	0.238	0.06	2.48E-189
HNRNPM	2.15E-193	0.472016534	0.899	0.749	4.54E-189
FBL	1.45E-192	0.41528047	0.598	0.33	3.06E-188
EMC9	2.42E-192	0.322252204	0.298	0.097	5.12E-188
CLGN	2.96E-192	0.306179375	0.324	0.11	6.25E-188
IDH2	2.72E-191	0.466403479	0.681	0.426	5.76E-187
SPDL1	1.24E-190	0.287461402	0.248	0.067	2.62E-186
COA4	2.52E-190	0.352608517	0.487	0.228	5.33E-186
PTPMT1	2.66E-189	0.287693593	0.321	0.11	5.62E-185
SNRPF	3.76E-189	0.42250012	0.919	0.782	7.96E-185
BCAN	4.76E-189	0.255678285	0.116	0.009	1.01E-184
SEPHS1	6.50E-189	0.321170817	0.345	0.126	1.38E-184
NIPSNAP2	8.93E-188	0.350525271	0.476	0.222	1.89E-183
H2AFJ	1.20E-187	0.254003476	0.238	0.062	2.53E-183
NUDT1	2.15E-187	0.482497122	0.639	0.392	4.54E-183
MMAB	2.76E-187	0.29639706	0.296	0.095	5.84E-183
ING5	1.19E-186	0.258418348	0.264	0.076	2.52E-182
MSI2	1.49E-186	0.452250876	0.625	0.354	3.16E-182
BAZ1A	2.62E-185	0.389302412	0.509	0.249	5.53E-181
AKAP12	3.35E-185	0.355630776	0.436	0.192	7.08E-181
SNRNP25	1.45E-184	0.347702562	0.461	0.214	3.07E-180
ITGB1	2.16E-183	0.343086305	0.517	0.253	4.56E-179
NUDC	5.19E-183	0.39566181	0.719	0.452	1.10E-178
ARHGAP5	4.33E-182	0.37551297	0.422	0.186	9.15E-178
RUVBL1	4.54E-182	0.351318114	0.473	0.224	9.60E-178
GAR1	5.34E-182	0.284496486	0.322	0.114	1.13E-177
GNL3	1.53E-181	0.349723078	0.413	0.177	3.23E-177

SDHAF3	3.78E-181	0.285195434	0.327	0.117	8.00E-177
RGS16	4.29E-181	0.399074092	0.195	0.042	9.07E-177
ACADM	1.17E-180	0.341540042	0.477	0.226	2.48E-176
CNTFR	1.51E-180	0.352140287	0.41	0.178	3.20E-176
HMGN1	1.77E-180	0.355658507	0.992	0.971	3.73E-176
POLR2L	1.38E-179	0.422024065	0.699	0.436	2.92E-175
BRIX1	1.75E-179	0.320625433	0.438	0.196	3.70E-175
PIM1	1.78E-179	0.25608666	0.245	0.068	3.77E-175
RFXANK	9.86E-179	0.276103256	0.295	0.098	2.08E-174
TCF3	2.17E-178	0.327419387	0.43	0.19	4.58E-174
HIBCH	2.18E-178	0.273061862	0.312	0.108	4.60E-174
NT5DC2	2.79E-178	0.37280822	0.482	0.239	5.89E-174
TMEM237	8.42E-178	0.297717041	0.383	0.155	1.78E-173
GLCCI1	1.15E-177	0.396810552	0.46	0.217	2.42E-173
HAUS6	1.46E-177	0.25804513	0.287	0.092	3.09E-173
IFT74	2.47E-177	0.251199137	0.223	0.057	5.23E-173
PLAGL1	6.32E-177	0.255966235	0.192	0.042	1.34E-172
FDX1	1.01E-176	0.291529926	0.338	0.126	2.14E-172
PPIL1	3.93E-176	0.29046919	0.394	0.164	8.31E-172
SSRP1	4.08E-176	0.36593996	0.547	0.289	8.64E-172
CDC42EP4	1.09E-175	0.327566536	0.415	0.181	2.30E-171
TMEM158	1.16E-175	0.419991995	0.324	0.122	2.46E-171
DKC1	1.02E-174	0.322724527	0.396	0.168	2.15E-170
EEF1E1	1.92E-174	0.341763692	0.491	0.242	4.06E-170
NRARP	2.38E-174	0.296982815	0.25	0.073	5.04E-170
PTN	3.47E-174	1.616693199	0.386	0.181	7.34E-170
RPL39L	2.24E-173	0.306892648	0.352	0.139	4.73E-169
PPIH	3.33E-173	0.296424249	0.4	0.17	7.03E-169
DDAH1	4.46E-173	0.271915578	0.231	0.063	9.43E-169
SMAD5	5.89E-173	0.330599875	0.397	0.17	1.24E-168
MPDZ	6.62E-173	0.2969895	0.318	0.113	1.40E-168
PMF1	1.10E-172	0.349189167	0.537	0.283	2.32E-168
SNHG16	1.31E-171	0.36409058	0.483	0.24	2.77E-167
CARHSP1	2.74E-171	0.479516401	0.78	0.582	5.79E-167
MSH6	4.96E-171	0.364066735	0.409	0.183	1.05E-166
RUVBL2	6.82E-171	0.326743641	0.443	0.206	1.44E-166
NDUFAF3	1.27E-170	0.351921973	0.517	0.267	2.68E-166
HADHB	1.60E-170	0.270580762	0.318	0.116	3.38E-166
APBB2	1.99E-170	0.307304675	0.368	0.15	4.22E-166
EIF2AK2	5.88E-170	0.286614635	0.338	0.128	1.24E-165
IGF2BP1	1.22E-169	0.358717256	0.498	0.251	2.58E-165
MYL12A	3.85E-169	0.293534922	0.441	0.202	8.13E-165
FERMT2	5.86E-169	0.274016289	0.34	0.13	1.24E-164
ALKBH2	1.50E-168	0.273429002	0.307	0.109	3.17E-164
HMGA1	3.99E-168	0.547051549	0.73	0.528	8.43E-164
VRK1	7.49E-168	0.333872487	0.442	0.203	1.58E-163
MRPL12	1.41E-167	0.304722004	0.391	0.168	2.98E-163
ACAT2	5.64E-167	0.468729987	0.619	0.371	1.19E-162
SDHD	8.38E-167	0.291685278	0.377	0.158	1.77E-162
SCCPDH	1.68E-166	0.319287574	0.461	0.22	3.56E-162
ASPH	1.93E-166	0.265697305	0.286	0.097	4.08E-162
SUZ12	2.22E-166	0.328823955	0.431	0.198	4.70E-162
PSME2	2.36E-166	0.294626763	0.345	0.136	4.99E-162
HINT2	2.73E-166	0.283819098	0.334	0.129	5.76E-162
RAD51C	4.73E-166	0.270244172	0.32	0.119	9.99E-162
MANF	1.44E-165	0.325149608	0.425	0.193	3.05E-161
NIFK	2.16E-165	0.362359168	0.505	0.26	4.56E-161
UNG	6.32E-165	0.319584569	0.301	0.111	1.34E-160

PSMC3	7.38E-165	0.409104606	0.742	0.51	1.56E-160
TSEN15	8.26E-165	0.259544552	0.312	0.113	1.75E-160
MOB1A	1.58E-164	0.279969099	0.347	0.137	3.34E-160
MOB3B	2.45E-164	0.269154935	0.283	0.096	5.18E-160
RPS9	3.62E-164	0.34512469	0.999	0.999	7.65E-160
HNRNPU	5.74E-164	0.478469182	0.911	0.804	1.21E-159
PRIM1	8.20E-164	0.267689577	0.297	0.105	1.73E-159
DLEU2	6.69E-163	0.294096885	0.303	0.11	1.42E-158
BRD7	3.40E-162	0.321496302	0.458	0.22	7.20E-158
HIST1H2BC	3.68E-162	0.294393539	0.167	0.034	7.78E-158
ACTL6A	6.60E-162	0.327372572	0.491	0.251	1.39E-157
COA1	5.85E-161	0.328109705	0.482	0.243	1.24E-156
PRDX3	2.86E-160	0.340759932	0.599	0.346	6.06E-156
IMPDH2	3.21E-160	0.357004873	0.507	0.268	6.80E-156
JAM3	3.56E-160	0.333987041	0.412	0.188	7.54E-156
RANGRF	8.95E-160	0.251117953	0.258	0.083	1.89E-155
HNRNPR	1.97E-159	0.396727192	0.937	0.848	4.17E-155
SPAG16	3.89E-159	0.314278752	0.358	0.15	8.22E-155
ABCD3	4.01E-159	0.276164871	0.323	0.124	8.49E-155
ZCRB1	2.48E-158	0.379862538	0.609	0.355	5.24E-154
HNRNPUL1	3.07E-158	0.366577637	0.579	0.332	6.50E-154
RAD23A	1.17E-157	0.342321519	0.5	0.264	2.47E-153
SLC25A5	4.09E-157	0.465658058	0.764	0.561	8.66E-153
NSD2	4.25E-157	0.322782596	0.433	0.206	8.99E-153
PUF60	5.00E-157	0.397095526	0.774	0.562	1.06E-152
TSPAN3	3.80E-156	0.377572568	0.744	0.507	8.04E-152
SNX17	2.36E-155	0.263896009	0.324	0.126	4.98E-151
PRXL2A	8.83E-154	0.376524217	0.59	0.337	1.87E-149
SNX3	1.82E-153	0.386697337	0.711	0.48	3.84E-149
SMC5	5.00E-153	0.262672325	0.305	0.115	1.06E-148
NAXE	2.26E-152	0.295219988	0.375	0.166	4.79E-148
CYC1	5.58E-152	0.344485741	0.656	0.409	1.18E-147
NDUFC1	1.11E-151	0.387349557	0.725	0.486	2.34E-147
IMMP1L	1.88E-151	0.314801654	0.436	0.211	3.98E-147
HIST2H2BF	3.64E-151	0.357920311	0.175	0.042	7.69E-147
RCC2	7.17E-151	0.271351525	0.35	0.146	1.52E-146
EIF4A3	1.01E-150	0.324307998	0.531	0.291	2.13E-146
CHIC2	1.83E-150	0.263973643	0.327	0.131	3.86E-146
MICOS10	2.72E-150	0.360771997	0.706	0.46	5.76E-146
C5orf30	3.16E-150	0.311922813	0.393	0.181	6.68E-146
POLR2G	5.04E-150	0.34187588	0.639	0.391	1.07E-145
RPS3	5.54E-150	0.317742045	0.999	1	1.17E-145
DNAJC19	6.27E-149	0.31356668	0.445	0.222	1.32E-144
TIMM10	1.26E-148	0.283952763	0.394	0.179	2.66E-144
FUS	2.33E-148	0.368670646	0.938	0.848	4.92E-144
KMT5A	1.34E-147	0.291123291	0.261	0.091	2.83E-143
POLR2J	1.50E-147	0.351279218	0.692	0.453	3.17E-143
ACYP1	3.58E-147	0.325299843	0.447	0.226	7.57E-143
FADS1	1.22E-146	0.359691739	0.596	0.351	2.58E-142
PPP1CA	1.30E-146	0.298803474	0.497	0.261	2.74E-142
ISYNA1	6.31E-146	0.294068489	0.37	0.163	1.33E-141
ARL4A	8.67E-146	0.263628463	0.28	0.101	1.83E-141
SCP2	5.67E-145	0.302908708	0.444	0.222	1.20E-140
PSD3	6.66E-145	0.296676	0.377	0.169	1.41E-140
TLE3	1.08E-144	0.269757407	0.291	0.11	2.28E-140
TOPBP1	2.16E-144	0.262402474	0.317	0.127	4.57E-140
DHX40	8.83E-144	0.319351488	0.432	0.213	1.87E-139
MLEC	3.67E-143	0.291789809	0.397	0.186	7.76E-139

MRPL23	4.22E-143	0.298901289	0.425	0.21	8.93E-139
TEAD1	8.94E-143	0.27922101	0.371	0.165	1.89E-138
PDIA4	1.42E-142	0.254079452	0.309	0.123	3.01E-138
ARL2	1.62E-142	0.295739598	0.4	0.187	3.42E-138
METTL5	2.26E-142	0.266263003	0.367	0.161	4.78E-138
ADH5	2.27E-142	0.364264913	0.72	0.488	4.81E-138
HACD3	2.82E-142	0.354349602	0.629	0.388	5.96E-138
KNOP1	7.24E-142	0.359289174	0.48	0.259	1.53E-137
DCP2	8.49E-142	0.288654214	0.355	0.156	1.79E-137
NUTF2	3.58E-141	0.316823217	0.528	0.295	7.57E-137
NSRP1	3.93E-141	0.326818013	0.411	0.199	8.32E-137
DECR1	6.09E-141	0.269254646	0.366	0.163	1.29E-136
TMEM230	1.75E-140	0.31299417	0.501	0.274	3.70E-136
HILPDA	2.01E-140	0.294890597	0.413	0.201	4.26E-136
SSNA1	2.63E-140	0.292140708	0.483	0.256	5.57E-136
ITGB1BP1	3.72E-140	0.327410889	0.509	0.282	7.87E-136
SNRPD2	3.46E-139	0.377003577	0.911	0.803	7.31E-135
IPO7	7.59E-139	0.292319628	0.413	0.2	1.60E-134
UQCRC1	7.90E-139	0.322089374	0.6	0.365	1.67E-134
UBE2S	1.02E-138	1.299834411	0.706	0.64	2.16E-134
ERCC1	2.27E-138	0.308184129	0.485	0.26	4.81E-134
SNRPE	2.76E-138	0.392368253	0.9	0.789	5.83E-134
BCCIP	4.89E-138	0.285946217	0.383	0.179	1.03E-133
SUPT16H	2.59E-137	0.391203951	0.64	0.411	5.47E-133
HMGCS1	3.89E-137	0.500362025	0.592	0.371	8.23E-133
FMC1	4.12E-137	0.299266258	0.494	0.264	8.70E-133
RIF1	1.98E-136	0.32190056	0.442	0.226	4.19E-132
MRPL40	2.25E-136	0.28575997	0.4	0.192	4.76E-132
TET1	8.74E-136	0.323623151	0.432	0.22	1.85E-131
FAM136A	9.81E-136	0.290090324	0.409	0.201	2.07E-131
SERPINH1	1.29E-135	0.271655331	0.209	0.062	2.73E-131
ACP1	1.48E-135	0.339258791	0.686	0.463	3.14E-131
ILF2	1.72E-135	0.404819762	0.842	0.693	3.64E-131
NDUFAF8	2.19E-135	0.273682119	0.412	0.201	4.64E-131
SKA2	4.35E-135	0.535303918	0.724	0.547	9.20E-131
SMC3	5.64E-135	0.44383635	0.815	0.658	1.19E-130
MSI1	2.08E-134	0.272717008	0.396	0.189	4.40E-130
PNKD	2.15E-134	0.298449313	0.462	0.245	4.54E-130
MBIP	2.16E-133	0.386259212	0.454	0.241	4.57E-129
MAZ	3.17E-133	0.416620229	0.698	0.491	6.71E-129
TMEM256	4.63E-133	0.319618085	0.466	0.252	9.79E-129
RNH1	6.09E-133	0.29335103	0.431	0.219	1.29E-128
UFD1	1.07E-132	0.292509743	0.431	0.221	2.26E-128
SMIM7	1.73E-132	0.29672312	0.519	0.292	3.66E-128
COX17	4.53E-132	0.303951834	0.496	0.271	9.58E-128
ZSWIM7	4.88E-132	0.257901705	0.345	0.153	1.03E-127
HIST1H2AK	5.51E-132	0.361538843	0.219	0.073	1.16E-127
AK6	9.92E-132	0.253904966	0.342	0.152	2.10E-127
SLF1	1.17E-131	0.294125005	0.358	0.159	2.48E-127
CASP8AP2	2.34E-131	0.278416681	0.336	0.148	4.94E-127
LSM8	1.19E-128	0.30865159	0.672	0.436	2.52E-124
MRPL13	1.09E-127	0.275685407	0.439	0.228	2.31E-123
EFNB1	1.37E-127	0.260950521	0.31	0.132	2.91E-123
ETFA	1.40E-127	0.262776227	0.376	0.18	2.96E-123
RPL17	3.46E-127	0.38490177	0.998	0.999	7.32E-123
SNRNP40	4.04E-127	0.269999722	0.444	0.233	8.54E-123
PSMA4	1.84E-126	0.351879393	0.767	0.561	3.89E-122
NDUFS6	8.76E-126	0.340036805	0.828	0.638	1.85E-121

KDELR2	1.43E-125	0.266684752	0.402	0.202	3.02E-121
RPL10A	5.17E-125	0.344022977	0.996	0.998	1.09E-120
RAB8A	1.05E-124	0.258561063	0.367	0.175	2.22E-120
COMT	1.09E-124	0.285096837	0.46	0.25	2.31E-120
MTHFD2L	1.20E-124	0.255045632	0.331	0.148	2.53E-120
PNN	1.20E-124	0.386537025	0.788	0.607	2.54E-120
CENPV	1.25E-124	0.463876921	0.743	0.56	2.64E-120
MRPL14	2.01E-124	0.29003012	0.513	0.297	4.25E-120
C7orf50	7.84E-124	0.297342877	0.487	0.272	1.66E-119
UBB	8.75E-124	0.347211563	0.993	0.988	1.85E-119
CYB5B	1.26E-123	0.266035547	0.395	0.197	2.67E-119
PSMG2	2.24E-123	0.273590803	0.466	0.254	4.75E-119
CCNG1	2.36E-123	0.250287964	0.318	0.14	4.99E-119
CEP70	2.68E-123	0.284120382	0.249	0.095	5.66E-119
RPS20	5.14E-123	0.394575506	0.98	0.94	1.09E-118
POLE4	1.34E-122	0.265418593	0.377	0.181	2.84E-118
RPS16	2.18E-122	0.296452499	0.999	0.998	4.61E-118
RAD23B	2.76E-122	0.271927242	0.429	0.226	5.84E-118
MRPS26	6.64E-122	0.308356362	0.555	0.337	1.40E-117
C4orf3	7.27E-122	0.295842634	0.559	0.334	1.54E-117
PPP1CC	1.18E-121	0.314514997	0.699	0.477	2.49E-117
KHSRP	4.79E-121	0.270159698	0.446	0.237	1.01E-116
CD81	5.20E-121	0.359470056	0.483	0.282	1.10E-116
NOL7	7.51E-121	0.320830776	0.645	0.429	1.59E-116
LRPAP1	2.07E-120	0.259599004	0.387	0.192	4.39E-116
HNRNPL	4.11E-120	0.297533285	0.551	0.334	8.68E-116
ODC1	1.25E-119	0.367054158	0.611	0.391	2.64E-115
PRELID1	1.67E-119	0.31977797	0.599	0.384	3.53E-115
OARD1	5.64E-119	0.256890771	0.433	0.227	1.19E-114
PPP2R3C	8.00E-119	0.265711044	0.444	0.239	1.69E-114
MRPL22	8.43E-119	0.263345226	0.458	0.25	1.78E-114
HIST1H2BF	2.08E-118	0.334838647	0.198	0.066	4.40E-114
CSTB	5.67E-118	0.294492422	0.596	0.373	1.20E-113
CANX	7.15E-118	0.32179971	0.535	0.322	1.51E-113
RNPS1	7.55E-118	0.298533474	0.599	0.381	1.60E-113
NOP58	9.14E-118	0.276673114	0.44	0.237	1.93E-113
CALR	1.97E-117	0.394452287	0.638	0.429	4.16E-113
POU3F3	4.11E-117	0.383472024	0.713	0.502	8.70E-113
ECI2	5.29E-117	0.263330345	0.39	0.197	1.12E-112
ESD	6.15E-117	0.313136098	0.716	0.5	1.30E-112
CBX5	8.80E-117	0.369082834	0.882	0.73	1.86E-112
CKAP5	6.22E-116	0.395080558	0.391	0.211	1.32E-111
MESD	7.59E-116	0.267946212	0.433	0.23	1.61E-111
CSE1L	7.79E-116	0.262025057	0.398	0.206	1.65E-111
TOMM6	9.28E-116	0.333234848	0.665	0.453	1.96E-111
MAPK1IP1L	3.12E-115	0.263946107	0.439	0.238	6.60E-111
EIF1AX	5.71E-115	0.370668234	0.786	0.615	1.21E-110
APRT	6.24E-115	0.324382672	0.494	0.292	1.32E-110
GRHPR	1.67E-114	0.274608522	0.478	0.272	3.53E-110
NKAIN4	1.74E-114	0.271582929	0.351	0.169	3.68E-110
CETN2	3.74E-114	0.317116067	0.468	0.264	7.90E-110
NNT-AS1	4.18E-114	0.262084806	0.378	0.189	8.84E-110
TMSB15A	6.19E-114	0.325766161	0.972	0.927	1.31E-109
NPAS3	1.75E-113	0.251334199	0.304	0.134	3.70E-109
SFTA3	3.38E-113	0.294150282	0.226	0.083	7.14E-109
PSMB3	8.03E-113	0.314702504	0.773	0.579	1.70E-108
MRTO4	3.73E-112	0.250080973	0.331	0.157	7.89E-108
RAB5IF	1.09E-111	0.261925561	0.508	0.297	2.31E-107

BORCS7	2.27E-111	0.256504669	0.39	0.201	4.79E-107
P4HB	5.01E-111	0.250516394	0.391	0.202	1.06E-106
PMVK	1.41E-110	0.258549192	0.373	0.189	2.97E-106
PHB	1.99E-110	0.302269964	0.606	0.395	4.21E-106
MAT2A	2.61E-110	0.278258599	0.445	0.247	5.52E-106
SPECC1	4.12E-110	0.252019387	0.358	0.177	8.72E-106
SLC25A3	1.16E-109	0.316240576	0.932	0.862	2.46E-105
DLK1	1.83E-107	0.706465346	0.148	0.041	3.86E-103
RAP1B	2.81E-107	0.251964554	0.449	0.249	5.94E-103
IDI1	3.12E-107	0.395164756	0.617	0.423	6.60E-103
PDCD5	1.60E-106	0.311979603	0.728	0.525	3.39E-102
PPIG	2.15E-106	0.316050149	0.741	0.537	4.55E-102
IGF2BP3	2.67E-106	0.252187372	0.381	0.197	5.65E-102
FKBP3	2.68E-106	0.330860923	0.807	0.633	5.67E-102
PAX6	4.35E-106	0.362746266	0.284	0.126	9.20E-102
SYNE2	5.54E-106	0.484521548	0.743	0.581	1.17E-101
THYN1	6.67E-106	0.271274385	0.497	0.295	1.41E-101
EBNA1BP2	7.78E-106	0.260102871	0.429	0.238	1.64E-101
PABPC1	2.79E-105	0.348213285	0.822	0.665	5.90E-101
MRPL52	1.41E-104	0.27705318	0.606	0.393	2.98E-100
CYCS	3.26E-103	0.308467373	0.74	0.549	6.90E-99
ABCF1	5.04E-103	0.254699135	0.413	0.224	1.07E-98
HMGXB4	2.02E-102	0.263020937	0.456	0.263	4.28E-98
SRSF9	2.61E-102	0.321717666	0.724	0.533	5.52E-98
PARD6G-AS1	1.91E-101	0.255395599	0.202	0.074	4.04E-97
ZNF22	2.31E-101	0.287860533	0.686	0.485	4.87E-97
FGD5-AS1	2.33E-101	0.268934861	0.487	0.29	4.93E-97
RBM17	4.09E-101	0.264821462	0.569	0.362	8.64E-97
MAGI1	4.32E-101	0.256196829	0.393	0.21	9.14E-97
GADD45GIP1	4.54E-101	0.303450327	0.682	0.481	9.59E-97
LYRM2	7.23E-101	0.266467745	0.508	0.306	1.53E-96
CFAP20	8.80E-101	0.259986817	0.514	0.316	1.86E-96
HNRNPH3	1.81E-100	0.315569557	0.907	0.825	3.83E-96
PCNP	2.02E-100	0.267576915	0.589	0.381	4.27E-96
XRCC6	4.87E-100	0.292061166	0.759	0.577	1.03E-95
POU3F2	5.23E-100	0.264110776	0.435	0.242	1.11E-95
PKN2	3.32E-99	0.265978981	0.465	0.274	7.02E-95
YBX1	2.44E-98	0.386580714	0.994	0.99	5.17E-94
AURKAIP1	3.35E-97	0.268073241	0.679	0.473	7.09E-93
HIST1H4E	7.03E-97	0.38832252	0.281	0.136	1.49E-92
EIF4EBP1	1.04E-96	0.285851255	0.472	0.283	2.21E-92
RPS17	1.95E-95	0.411220444	0.964	0.923	4.12E-91
CHD7	2.16E-95	0.269862867	0.485	0.289	4.57E-91
DAD1	3.54E-95	0.275296428	0.688	0.484	7.48E-91
CTBP2	4.08E-95	0.253404611	0.518	0.316	8.62E-91
EIF5A	8.90E-95	0.284381443	0.686	0.491	1.88E-90
SQLE	1.73E-94	0.356041782	0.697	0.515	3.66E-90
BAG1	1.92E-94	0.258985288	0.421	0.242	4.06E-90
CCAR1	2.64E-94	0.288816892	0.574	0.375	5.58E-90
TPR	8.30E-93	0.310511823	0.592	0.401	1.75E-88
PSMB1	1.01E-92	0.291171312	0.819	0.657	2.13E-88
RHOBTB3	1.50E-91	0.285969395	0.538	0.351	3.16E-87
TRIM28	1.62E-91	0.264944124	0.591	0.393	3.42E-87
FXR1	4.05E-91	0.280741985	0.664	0.467	8.56E-87
DHX9	7.99E-91	0.26755623	0.559	0.365	1.69E-86
TIMM13	8.04E-91	0.292999152	0.609	0.423	1.70E-86
SET	1.89E-90	0.32385583	0.949	0.896	4.00E-86
COX8A	1.03E-89	0.319606564	0.905	0.838	2.18E-85

SPAG9	6.19E-89	0.290352962	0.586	0.398	1.31E-84
SF1	3.74E-88	0.263219631	0.653	0.456	7.92E-84
PSMA5	1.52E-87	0.254005854	0.513	0.329	3.21E-83
LARS	2.38E-87	0.255439963	0.464	0.282	5.04E-83
UBXN4	6.04E-87	0.27201462	0.696	0.513	1.28E-82
CCT6A	2.13E-86	0.281877595	0.777	0.607	4.51E-82
NDUFB2	7.56E-86	0.281561098	0.815	0.651	1.60E-81
GIHCG	1.86E-85	0.258418685	0.381	0.216	3.94E-81
PSMB6	6.27E-85	0.269284535	0.837	0.686	1.33E-80
PSMD7	1.09E-84	0.251680261	0.658	0.464	2.30E-80
TCEA1	1.45E-84	0.257417224	0.644	0.452	3.07E-80
CDKN1B	1.66E-84	0.283236736	0.572	0.391	3.50E-80
ZBTB20	2.42E-84	0.282124307	0.589	0.397	5.13E-80
SOD1	2.52E-84	0.303248262	0.815	0.663	5.34E-80
NHP2	2.58E-84	0.289087206	0.7	0.522	5.45E-80
CACYBP	3.05E-84	0.256773448	0.75	0.553	6.45E-80
SSBP1	2.86E-83	0.268465257	0.762	0.589	6.05E-79
MZT2A	9.25E-83	0.257603257	0.578	0.391	1.96E-78
RPS18	1.31E-82	0.296379344	0.999	1	2.77E-78
TCEAL9	2.46E-82	0.286103026	0.702	0.504	5.20E-78
ROMO1	5.44E-82	0.252947196	0.699	0.51	1.15E-77
TMEM258	5.02E-79	0.268021798	0.777	0.618	1.06E-74
HIGD2A	9.44E-79	0.257285117	0.627	0.439	2.00E-74
PRPF40A	3.80E-78	0.258383144	0.655	0.472	8.03E-74
PCBP2	1.99E-77	0.277147193	0.863	0.737	4.20E-73
FAM171B	2.74E-75	0.261166866	0.301	0.163	5.80E-71
HSPA5	3.07E-75	0.274827795	0.478	0.311	6.49E-71
EIF3M	3.15E-75	0.274506442	0.695	0.531	6.66E-71
KTN1	2.11E-73	0.25129413	0.575	0.394	4.45E-69
EID1	2.73E-73	0.250257507	0.978	0.943	5.77E-69
CENPX	1.53E-72	0.274292826	0.578	0.416	3.23E-68
RPS14	1.89E-72	0.2606568	0.999	1	3.99E-68
MGST3	5.96E-71	0.268319271	0.662	0.485	1.26E-66
DDIT4	3.53E-69	0.28148974	0.502	0.338	7.47E-65
G2E3	2.61E-67	0.312905944	0.35	0.216	5.53E-63
NTRK2	2.71E-67	0.288799818	0.21	0.1	5.73E-63
MIF	9.08E-64	0.273723798	0.965	0.954	1.92E-59
PHPT1	2.46E-63	0.252966314	0.746	0.593	5.21E-59
PPA1	2.63E-62	0.267034803	0.835	0.734	5.56E-58
SAP30	4.23E-61	0.254411477	0.325	0.196	8.94E-57
DYNLL1	3.31E-57	0.27133703	0.969	0.957	7.01E-53
CYP51A1	2.86E-56	0.263058983	0.44	0.298	6.05E-52
SFPQ	4.96E-54	0.281252753	0.841	0.745	1.05E-49
DNAJB1	3.17E-50	0.42156868	0.649	0.491	6.69E-46
HIST1H2AE	4.48E-47	0.252394408	0.241	0.143	9.47E-43
TPM1	5.55E-26	0.317061517	0.42	0.335	1.17E-21
H1FX	3.87E-11	0.250965781	0.802	0.732	8.18E-07
ARL6IP1	2.20E-09	1.19901714	0.698	0.771	4.66E-05
GADD45G	1.82E-08	0.484443927	0.391	0.368	0.000384789
C1orf61	0.294185639	0.299297529	0.839	0.853	1
